# The Societal Costs and Benefits of Commuter Bicycling: Simulating the Effects of Specific Policies Using System Dynamics Modeling

**DOI:** 10.1289/ehp.1307250

**Published:** 2014-02-04

**Authors:** Alexandra Macmillan, Jennie Connor, Karen Witten, Robin Kearns, David Rees, Alistair Woodward

**Affiliations:** 1 School of Population Health, University of Auckland, Auckland, New Zealand; 2 Department of Preventive and Social Medicine, University of Otago, Dunedin, New Zealand; 3 Social and Health Outcomes Research and Evaluation (SHORE), Massey University, Auckland, New Zealand; 4 School of Environment, University of Auckland, Auckland, New Zealand; 5 Synergia Ltd, Auckland, New Zealand

## Abstract

Background: Shifting to active modes of transport in the trip to work can achieve substantial co-benefits for health, social equity, and climate change mitigation. Previous integrated modeling of transport scenarios has assumed active transport mode share and has been unable to incorporate acknowledged system feedbacks.

Objectives: We compared the effects of policies to increase bicycle commuting in a car-dominated city and explored the role of participatory modeling to support transport planning in the face of complexity.

Methods: We used system dynamics modeling (SDM) to compare realistic policies, incorporating feedback effects, nonlinear relationships, and time delays between variables. We developed a system dynamics model of commuter bicycling through interviews and workshops with policy, community, and academic stakeholders. We incorporated best available evidence to simulate five policy scenarios over the next 40 years in Auckland, New Zealand. Injury, physical activity, fuel costs, air pollution, and carbon emissions outcomes were simulated.

Results: Using the simulation model, we demonstrated the kinds of policies that would likely be needed to change a historical pattern of decline in cycling into a pattern of growth that would meet policy goals. Our model projections suggest that transforming urban roads over the next 40 years, using best practice physical separation on main roads and bicycle-friendly speed reduction on local streets, would yield benefits 10–25 times greater than costs.

Conclusions: To our knowledge, this is the first integrated simulation model of future specific bicycling policies. Our projections provide practical evidence that may be used by health and transport policy makers to optimize the benefits of transport bicycling while minimizing negative consequences in a cost-effective manner. The modeling process enhanced understanding by a range of stakeholders of cycling as a complex system. Participatory SDM can be a helpful method for integrating health and environmental outcomes in transport and urban planning.

Citation: Macmillan A, Connor J, Witten K, Kearns R, Rees D, Woodward A. 2014. The societal costs and benefits of commuter bicycling: simulating the effects of specific policies using system dynamics modeling. Environ Health Perspect 122:335–344; http://dx.doi.org/10.1289/ehp.1307250

## Introduction

Car use is the dominant mode of transport to work in many high-income cities. In car-oriented cities, commuting by private motor vehicle allows access to employment and training (crucial social determinants of health) while enabling households to manage competing responsibilities. However, car-dependent commuting has significant negative public health effects for commuters, the wider community, and local and global ecosystems. A mode shift to greater use of active transport would bring environmental, health, social, and equity benefits (de Nazelle et al. 2011; Hosking et al. 2011). In high-income cities, car commutes tend to be short, habitual, solitary trips in congested traffic. Consequently, they make a greater contribution to road traffic injury (Bhalla et al. 2007), air pollution and transport greenhouse gas emissions (André and Rapone 2009), noise (Hänninen and Knol 2011), and stress (Jansen et al. 2003) than other kinds of light vehicle trips. Traffic congestion at peak commute times is also a significant influence on constructing new road capacity with land use, environmental, and social costs (Coffin 2007; Fahrig 2003). In addition, the predictability of car commuting routes may make these trips amenable to a wider range of policy alternatives.

Previous integrated health impact assessments, based on existing evidence, suggest that a shift to human-powered transport modes (bicycling, walking, running, wheelchair, skating) for short commute trips would be good for health, aside from the risk of road traffic injury (Hosking et al. 2011; Woodcock et al. 2009). These trips incur negligible greenhouse gas and air pollution costs, incorporate physical activity into people’s daily lives, and cost little (potentially increasing equitable access to jobs) (Hosking et al. 2011). Although transport policy is identified as a determinant of the global noncommunicable disease (NCD) crisis, transport interventions have not been included in the United Nations’ priority actions for NCDs because of poor evidence of cost-effectiveness (Beaglehole et al. 2011). Previous comparative risk assessments have been undertaken for transport policy, climate change, and health (e.g., Lindsay et al. 2011; Rojas-Rueda et al. 2011; Woodcock et al. 2009). However, these assessments have been unable to directly compare specific policies seeking to increase active transport, and have not incorporated recognized system feedbacks (Woodcock et al. 2009).

The relationships between urban planning and health are complex, and evidence for them varies widely in source and quality. Furthermore, policies may trade gains made against some objectives at the expense of others. However, a set of methodological recommendations is emerging in the literature that might promote effective policy decisions in such complex systems. They include a systems approach; transdisciplinarity (integrating knowledge across policy, community, and the academy); community participation in decision making; and a focus on social justice and environmental sustainability (Charron 2012). There have been recent calls for complex systems research to tackle deep-seated problems such as physical inactivity (Kohl et al. 2012), obesity (Swinburn et al. 2011), and improving the contribution of urban planning to health (Rydin et al. 2012).

We used the principles above to develop a commuter cycling and public health model integrating physical, social, and environmental well-being. We used this model to identify cost-effective transport policies for improving public health. Auckland, New Zealand’s largest and fastest growing city (population ~ 1.5 million), was the case study.

A program of motorway development and low-density urban growth in Auckland has led to exponential growth in car ownership and use, with a collapse in use of public transport and bicycling as modes of transport (Mees 2010). Private motor vehicles are used for > 75% of commutes, and bicycles 1%. Recently, Auckland’s regional government has been promoting bicycling for transport to reduce motor vehicle use.

## Study Design

We used participatory system dynamics modeling (SDM) to involve community, academic, and policy stakeholders in a process that explored the dynamic effects of realistic policies. SDM is based on the following principles (Richardson 2011):

Complex systems include many interacting variables that change over time.
This pattern of interaction is a key driver of system behavior over time.
Interaction between variables is characterized by reinforcing (positive) loops, which amplify dynamic system patterns of behavior and balancing (negative) feedback loops, which dampen these patterns.
Complex systems are also characterized by the accumulation of “stocks” that could include people, information, or material resources.
Time is an important component of complex systems, and the pattern of cause-and-effect relationships may change variables at different rates over time, creating tensions between short- and long-term policy effects.

A system dynamics simulation model consists of a set of integral equations whose solutions are approximated to demonstrate dynamic system behavior, enabling curves of trends over time in outcomes of interest to be explored and compared for future policy options. Participatory SDM has been successfully used to improve decision making for a variety of difficult public health problems, including health system capacity to deal with chronic disease (Hirsch et al. 2010), infectious disease epidemiology (Atun et al. 2005), substance abuse prevention (Holder and Blose 1987), and obesity (Abdel-Hamid 2003). The method has also been used to consider the outcomes of transport policies on air quality (Stave 2002). As with most SDM efforts, these examples aimed to provide insights about the dynamic effects of policy alternatives by relating these back to the system structure, rather than attempting to make falsely precise absolute predictions about future outcomes in the context of complexity.

For the present study, we used a combination of primary and secondary data to develop a qualitative set of feedback loops. We used a purposive sampling strategy to identify 16 people who represented groups designing, influencing, or affected by transport policy, with the latter including groups likely to incur health inequities as a result of transport policies (see Supplemental Material, Table S1). We undertook semistructured interviews using cognitive mapping, a technique designed to elicit the implicit causal theories of interviewees, linking perceptions and behaviors around a particular question, issue, or system (Eden and Ackermann 2004).

We developed a preliminary set of feedback loops from these interviews by piecing together the relationships identified in the individual interviews and triangulating the interview data with a multidisciplinary literature review about transport and public health. The focus was on refuting or supporting the dynamic causal theory emerging from the interviews. Although it was not within the scope of the study to undertake systematic reviews for each relationship identified, we used the interview data together with an ecosystem health framework (Hancock 1993), describing the relationships among health, equity, and sustainability, to guide a wide search of the health, transport, social sciences, and geography literatures. The preliminary feedback loops were refined in two SDM workshops as well as in meetings with individuals and organizations involving > 30 stakeholders. These workshops and meetings involved those already interviewed and other representatives of their group or organization. This resulted in a minimum set of feedback loops that represent a collective causal theory to explain trends over time for commuting in Auckland, including a causal loop diagram specific to commuter bicycling. This step was undertaken using Vensim PLE® modeling software (Ventana Systems UK, Salisbury, UK).

We used the bicycling causal loops to develop a simulation model, populating the variables and relationships in the diagram with best available epidemiological evidence, administrative data, and expert opinion when no data were available. Data sets included regional subsets of national census and survey data and Auckland-specific travel surveys. We combined regional survey data about transport preferences with the qualitative data from the interviews and workshops to quantify relationships between factors that influence transport mode behavior in the trip to work, and the resulting commuting mode shares. It was beyond the scope of this study to undertake separate systematic reviews for each relationship identified. For the relationships between changes in commuting and changes in well-being outcomes, we used existing systematic reviews of the literature, as well as regionally relevant relationships previously developed from systematic reviews. In choosing between sources of data, we considered multiple dimensions including epidemiological quality, relevance to the population being modeled, and completeness and quality of survey and administrative data. We used STELLA® (http://www.iseesystems.com) to simulate the model. Outcomes simulated were commuter cyclist injury, regional air pollution mortality and morbidity, physical activity–related mortality, greenhouse gas emissions, and individual fuel savings. We incorporated expected trends in population growth, all-cause mortality, public transport improvement, light vehicle fleet composition, and fuel price.

We triangulated the bicycling causal loop diagram with studies about the effectiveness of interventions to increase commuter bicycling to develop policy scenarios that might be most likely to alter the shape of trends in cycling over time. These were compared against a “business-as-usual” nonintervention scenario and the investment in cycling proposed in the region’s 30-year strategic transport plan (Auckland Regional Council 2010). We assessed the effectiveness of policy scenarios against strategic targets set out in the plan (Table 1). The model was run from 1991 through 2051. Historical simulations (1991–2012) were compared with the model simulations, validating the shape and magnitude of simulated outputs against existing data. Implementation of interventions was simulated gradually across the region during the simulation period, according to S-shaped implementation curves, beginning in 2012. The Regional Cycle Network (Auckland Regional Council 2010) is expected to be “complete” by 2040. Further scenarios were modeled to be complete by 2050.

**Table 1:** Transport strategic targets relevant to commuter bicycling from the Auckland Regional Land Transport Strategy 2010–2040 (Auckland Regional Council 2010).

Strategic objective	Current level	Quantified target for 2040
Road traffic injury	2005–2007 average: 74 deaths and 537 serious injuries	Reduction in road deaths to ≤ 40/year and serious injuries to ≤ 288/year
Congestion on the road freight network	2006–2009 average delay: 0.53 min/km	No increase
Walking and bicycling mode share	2010: walking 14%, bicycling < 1%	Combined walking and cycling mode share of 35%
Perception of bicycling safety	2010: 19% of survey respondents considered bicycling to be “always or mostly” safe	80% of people consider bicycling “always or mostly” safe
Transport greenhouse gas emissions	2007: 3.1 metric tons per capita from commuting	Halve per capita emissions from domestic transport compared with 2007
Current levels were reported in the Strategy, except 2007 commuting greenhouse gas emissions, which were simulated using VEPM 5.0 (Auckland Regional Council 2011).		

We reported scenario simulations as dynamic outcome curves that demonstrate the comparative direction, shape, and relative order of magnitude change in outcomes over time, in keeping with the reflective purpose of SDM. To make the findings policy relevant, we used the national transport agency’s methods (NZ Transport Agency 2010) to calculate indicative benefit–cost ratios in New Zealand dollars ($NZ) for each scenario compared with baseline (summed net benefits divided by infrastructure costs). Infrastructure costs were not inflated, and, in contrast to the transport agency’s methods, neither did we discount future benefits and harms. We used the national transport agency’s estimates (NZ Transport Agency 2010) to monetize bicycling fatal ($NZ3.1 million) and serious ($NZ0.325 million) injuries and the cost of greenhouse gas emissions ($NZ40/metric ton). We used the estimate for bicycling fatal injury to value mortality savings from reducing physical inactivity because these have not previously been monetized. We used the costs developed in Auckland regional air pollution modeling to monetize air pollution morbidity and mortality costs: premature mortality ($NZ0.75 million), cardiovascular and respiratory hospitalization (weighted average $NZ0.003 million), chronic obstructive pulmonary disease (COPD) hospitalization ($NZ0.075 million), cancer ($NZ0.75 million), and restricted activity days ($NZ98 per day) (Kuschel and Mahon 2010).

Establishing the validity of a system dynamics model involves a continuous process of building confidence in its utility as well as in its structural validity (Barlas 1996; Ford 2010; Forrester and Senge 1980; Schwaninger and Grösser 2008; Sterman 2000; van den Belt 2004; Vennix 1996). The first dimension included ensuring that the participatory process included appropriate stakeholders; that it balanced comprehensiveness (enough detail) with comprehensibility (ability to be understood); that it identified policy insights related back to the feedback structure; and that it influenced policy direction in the long term (Schwaninger and Grösser 2008). The second dimension included formal validity tests that were undertaken to assess the structural validity of the model (see Supplemental Material, Table S2). Sensitivity testing of both structural and parametric uncertainty was undertaken, including testing of opposing feedback theories about the effect of cyclist numbers on bicycling injuries against available data. In keeping with the reflective purpose of the model, and acknowledging that there would be significant uncertainty in the data, sensitivity analysis was based on a hierarchy of uncertainty effects: changes in parametric assumptions that altered the shape of behavior curves over time (e.g., changing growth into decline) were considered the most important to identify; variations that changed the order of magnitude of outcomes, but not the shape of behavior, were of secondary importance; and least significant were assumptions that altered the outcomes within an order of magnitude (Barlas 1996; Schwaninger and Grösser 2008). We tested the sensitivity of policy simulations using best- and worst-case assumptions, as well as a Monte Carlo approach to randomly sample from distributions of the most uncertain variables (see Supplemental Material, Table S3).

The study had ethics approval from the University of Auckland Human Participants Ethics Committee, and all participants gave informed consent before taking part.

## Results

**Figure 1:**
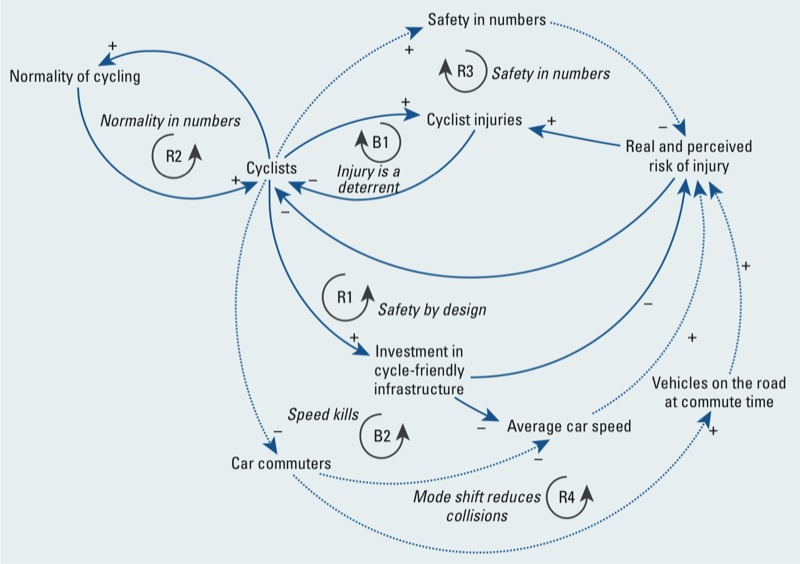
Causal loop diagram for bicycle commuting developed from stakeholder interviews and workshops, literature review, and data incorporation. Dotted lines denote loops identified by stakeholders and the literature, but where local data suggests they are currently inactive. Arrows with a positive sign (+) indicate that a change in the originating variable leads to a corresponding change in the variable at the arrowhead. Arrows with negative signs (–) indicate that a change in the originating variable leads to a change in the opposite direction for the arrowhead variable (R, reinforcing or positive feedback loop; B, balancing or negative feedback loop).

*Causal loop diagram*. The causal loop diagram was designed to provide a system explanation for the current trend in commuter cycling in Auckland (low-level oscillation about an equilibrium level of ~ 1% mode share), as well as assist with identifying policy levers to turn this trend into sustained growth in the future. The causal loop diagram for cycle commuting, emerging from the interviews and workshops, and triangulated with the literature, is shown in Figure 1 and consists of two balancing (B) and three reinforcing (R) feedbacks. It incorporates both structural and behavioral determinants of commuter cycling (Gatersleben and Appleton 2007). The first (B1) describes what is considered to be the dominant feedback loop acting in Auckland. With very little change in bicycling infrastructure, more people bicycling to work results in more bicycling crashes. These deaths and injuries appear to be the strongest psychological barrier to bicycling in high-income, car-dominant cities such as Auckland (Pooley et al. 2010; Winters et al. 2010), influencing perceived risk of injury (Kingham et al. 2011) and reducing bicycling. A reinforcing feedback loop (R1) describes the political influence that more cyclists can have on policy—increasing investment in safer bicycling facilities and encouraging bicycling. A further reinforcing loop (R2) indicates that more cycling to work can influence the behavior of other road users toward cycling, especially by demonstrating that cycling is possible across social groups without expensive equipment or extra training (Pucher and Buehler 2008).

Further feedbacks are possible at higher bicycling levels, but these were found to be currently very weak or inactive in Auckland during the incorporation of local data and validity testing. These are shown in dotted lines in Figure 1. In an alternative theory to B1, a “safety in numbers” loop is described in R3 (more people bicycling leads to a reduction in the rate of cyclist injuries). There is ecological evidence for a safety in numbers effect for bicycling (Jacobsen 2003; Tin Tin et al. 2011; Vandenbulcke et al. 2009), but this very likely combines the impact of safer infrastructure on cycling numbers and a direct effect of cyclist numbers on crash rates (Bhatia and Wier 2011; Wegman et al. 2010). We tested this theory using longitudinal bicycling mode share (census) and bicycling injury data (NZ Transport Agency 2005) for Auckland as part of the model validity testing. From this testing, we considered it unlikely that a “safety in numbers” effect occurs at Auckland’s low bicycling mode share, and that a threshold is likely for activation. Further positive feedback is possible with a significant mode shift from cars to bicycles at commute time, because reduced vehicle numbers reduce the likelihood of collision as well as improving perception of safety (R4, “mode shift reduces collisions”). Alternatively, a balancing loop (B2) is possible if a significant shift from cars to bicycles at peak times results in faster vehicles and increased real and perceived risk of bicycling injury, deterring cyclists. However, there is little evidence that this balancing loop applies on urban roads (Quddus et al. 2009).

The causal loop diagram is helpful for identifying the likely determinants of dynamic system behavior (e.g., trends over time in cyclist numbers or injuries). Other factors, such as weather and topography, which do not take part in the commuter bicycling feedback loops, are considered to be “exogenous.” Although they may moderate the level of outcomes, they are unlikely to influence the shape of trends over time. This feedback understanding influenced the policies chosen for simulation.

*The simulation model*. The simulation model incorporates the feedbacks described above. We developed mathematical relationships between variables from the best available data, and created nonfeedback structures to simulate exogenous outcomes such as physical activity. A brief summary of the assumptions and data sources for each sector is provided here. The full set of equations to develop the model is provided in Supplemental Material, Simulation Model Equations, pp. 20–44.

Commuting population growth. We used national and regional census-based statistics, projections of population growth, and labor force statistics (Statistics New Zealand 2009), assuming that the number of commuters would continue to grow at the same rate as the whole population, growing 40% over the simulation period from a baseline of 400,000 people. Sex, age, and ethnicity distributions for the commuting population were assumed to be stable. Commuters excluded those not traveling to work on census day. The noncommuting proportion of the working population was assumed to continue to be the same as historical (0.85).

Commute mode share. The New Zealand census includes a question about the main mode of travel used to get to work on census day. Analysis of data from the 1991 and subsequent censuses was used to populate baseline mode share levels for four main modes (light vehicle, bicycle, walking, and public transport) as well as helping to understand the effects of different influences over time. Because of the form of the census question, we assumed that commute trips are made by a single “main mode” and that the question asked on census day represents regular daily commuting patterns in the population. Baseline commute mode shares were as follows: light vehicle, 0.85; cycling, 0.02; walking, 0.055; public transport, 0.075. The annual number of vehicle kilometers traveled (VKT) by commuting via light vehicles is calculated from the light vehicle mode share, accounting for average commuting vehicle occupancy (because a proportion of light vehicle commuters are passengers), annual trips (accounting for part and full time employment), and the median light vehicle commute trip length. Consistent with the qualitative interviews, workshops, and stated choice transport models (Louviere et al. 2000), we used a relative utility structure to model the influences on mode share. We used a combination of the biennial local government transport mode rating surveys (Auckland Regional Council 2000–2010), census data about trip distance (Goodyear and Ralphs 2009), the stakeholder workshops, and qualitative studies about cycling (Daley et al. 2008; Kingham et al. 2011; Pooley et al. 2010; Pucher and Buehler 2008; Winters et al. 2010) to develop relationships between changes in population perception of different transport modes and changes in transport mode share. In doing so, we assumed that stated preference and revealed mode share were related and that there would be a delay (of 1 year on average) between a change in stated preference and a change in revealed mode share (Cantillo et al. 2007). We also assumed that trip distances of ≤ 6 km (50% of commute trips) were cycling range and trips of ≤ 2 km (27% of commute trips) were in walking range. These proportions were assumed to remain stable over the simulation period.

Bicycling injury. The structure simulating cyclist injury was particularly important because actual and perceived bicycling safety was central to many of the proposed feedback loops. We simulated cyclist serious injuries and deaths caused by a collision with a light vehicle. Almost all serious bicycling injuries in New Zealand involve a motor vehicle (Turner et al. 2009), and those involving a light vehicle are amenable to changes in commuting patterns. Various denominators are used in measures of cycling injury, and all have limitations depending on the research question (Halperin 1993). We chose a denominator that we postulated might reflect how individuals periodically consider the risk of bicycling to work in the area. Because we were considering changes in short commute trips from one mode to another, accounting for trip distance was less important. We therefore used a rate of injury per 1,000 cyclists. We used a simple simulation model to compare Auckland’s longitudinal cycling injury data with modeled data that included a safety-in-numbers effect in keeping with the power functions suggested by ecological studies (Jacobsen 2003; Robinson 2005; Tin Tin et al. 2011; Vandenbulcke et al. 2009). We found that simulations including a safety-in-numbers effect worsened the fit between modeled and historical data. Further, the longitudinal data suggested a reduction in the injury rate with declining cyclist numbers. This is in keeping with a recent critical review that suggests a threshold is likely and that a significant proportion of the safety in numbers effect seen in ecological studies is a “numbers-in-safety effect” (more safety because of better infrastructure, leading to more bicycling) (Bhatia and Wier 2011). We therefore included a threshold for safety in numbers at a mode share of 0.025, followed by a safety-in-numbers effect half that of Jacobsen’s power function.

Five steps were used to simulate fatal and serious cyclist injuries, adapted from the approach of Bhalla et al. (2007) and Elvik (2009):

Estimation of baseline proportions of vehicles and cyclists on local and main (arterial) roads, using Canadian evidence (Aultman-Hall et al. 1997) and local expert opinion;
Baseline annual collision and fatal or serious injury rates at commute time in the working-age population were developed through detailed analysis of the NZ Transport Agency Crash Analysis System, which includes information about timing, travel mode, demographics, injury categorization (minor, serious, and fatal), and crash environment (NZ Transport Agency 2005). This included adjustment for known underreporting of serious bicycling injuries in New Zealand (Alsop and Langley 2001; Christchurch Cycle Safety Committee 1991; Turner et al. 2006);
Calculation of the number of annual cyclist–car collisions accounting for the nonlinear impact of light vehicle numbers on local and arterial road collisions (Turner et al. 2009) and our modified safety-in-numbers effect;
Calculation of the number of fatal and serious injuries accounting for the nonlinear effect of changing mean car speeds on each type of road, using Auckland measurements of mean speeds (Beca Infrastructure Ltd 2011; Charlton et al. 2010). We adjusted accepted cumulative frequency curves [Organisation for Economic Co-operation and Development (OECD) and European Conference of Ministers of Transport 2006; Rosén et al. 2011] to differences between impact and mean speeds, and calibrated to Auckland speed and cyclist injury rates;
A combined serious and fatal injury rate per 1,000 cyclists was then calculated accounting for changes in the number of bicycle commuters.

To complete the feedback loops (R1, R2, and B1), we simulated a nonlinear impact of fatal and serious cyclist injury numbers on bicycling sense of safety supported by evidence from qualitative studies in bicycling environments similar to Auckland (Daley et al. 2008; Kingham et al. 2011; Pooley et al. 2010; Winters et al. 2010), using expert opinion to develop the shape of the curve relating media-reported deaths to perception of safety and calibrating it to Auckland fatal injury and transport mode rating surveys (Auckland Regional Council 2000–2010).

Air pollution, fuel cost savings, and greenhouse gas emission outcomes. Region- and source-specific estimates of air pollution mortality and morbidity are available from the Health and Air Pollution in New Zealand (HAPiNZ) (Fisher et al. 2007; Kuschel and Mahon 2010) models, which combine surrogate estimates of exposure from vehicle kilometers traveled, meteorological data, and spatial mapping (Kingham et al. 2007) with the effect estimates from international cohort studies. Outcomes in adults > 30 years of age estimated include deaths, cardiovascular and respiratory hospitalizations due to fine particulates (particulate matter ≤ 10 μm; PM_10_) and carbon monoxide, COPD hospitalizations due to PM_10_, cancer incidence due to benzene, and restricted activity days due to PM_10_. Baseline (1991) burden of disease metrics were adjusted for population, VKT (Ministry of Transport 2011), and vehicle fleet emissions changes over time. Auckland’s Vehicle Emissions Prediction Model (VEPM 5.0) (Auckland Regional Council 2011) and longitudinal emissions measurements (Bluett et al. 2011) were used to develop business-as-usual trends in PM_10_ and carbon monoxide. Dynamic air pollution burden of disease attributable to commuting was then simulated, accounting for changes to mode share, population, and light vehicle fleet emissions. By using the HAPiNZ modeling to calculate the burden of disease attributable to commuting, we have assumed that removing commuting light vehicles has an effect on air pollution equivalent to removing any light vehicle over a 24-hr period. The VEPM 5.0 model assumes that the New Zealand light vehicle fleet will follow European patterns of fuel efficiency and technical improvements, with a 10-year lag. We have also assumed that the health effects for commuters themselves of shifting travel mode are minimal because of the healthy worker effect, despite possible changes in exposure (de Hartog et al. 2010; Kaur et al. 2007).

A similar approach was taken to estimating the per VKT greenhouse gas emissions. VEPM 5.0 includes historical and projected carbon dioxide and carbon monoxide (also a potent greenhouse gas) vehicle emissions, accounting for changes in fuel consumptions, fuel composition, and vehicle technical improvements. Per VKT metrics were also estimated for methane and nitrous oxide using New Zealand’s Greenhouse Gas Inventory (Ministry for the Environment 2011). All these gases were converted to carbon dioxide equivalents (CO_2eq_) using the Fourth Intergovernmental Panel on Climate Change report (Forster et al. 2007). The model therefore simulates CO_2eq_ from the commuting fleet, accounting for trends in light vehicle emissions, population, and VKT. These are then converted to commuting CO_2eq_ and per capita emissions for the region’s population.

The direct fuel savings for average light vehicle commute trips averted were also calculated using VKT, accounting for fuel consumption and fleet composition trends (VEPM 5.0) and historical and forecast “at the pump” fuel costs for petrol and diesel (Donovan et al. 2009; Energy Information and Modelling Group 2009, 2011).

Mortality due to physical inactivity. We used two international prospective cohort studies of commuter bicycling (Andersen et al. 2000; Matthews et al. 2007) to develop a relative risk estimate for all-cause mortality of 0.72 for regular commuter cyclists. We assumed that the question about commute mode on census day represented the regular mode used for commuting, and that trips of ≤ 6 km in Auckland are equivalent to the levels of physical activity reported for commuter bicycling in the cohort studies. We also assumed a linear dose–response relationship for the range of commuter bicycling trips undertaken in Auckland. Lead time has not been specifically studied, and expert judgment has previously been used (Kahlmeier et al. 2011). We assumed a 2-year lead time for the accrual of physical activity benefits following a mode shift, and the same time for benefits to return to baseline with a reduction in commuter bicycling. To simulate all-cause mortality savings from any increase in commuter bicycling, we stratified the commuting population by age, ethnicity, and sex and projected and developed standardized baseline estimates of all-cause mortality in these groups from national mortality statistics. We used historical rates of declining all-cause mortality and projected these to develop an adjustment for the widespread trend in mortality. We simulated a “business-as-usual” all-cause mortality scenario, and then simulated all-cause mortality rates in response to any changes in commute mode share to calculate lives lost or saved due to changes in bicycle commuting.

Policy simulations. We investigated five policy scenarios (Table 1). The first scenario acted as a baseline (no investment in cycling), the second was drawn from present policy, and three further scenarios were developed by analyzing Auckland’s road network (Auckland Transport 2011; Charlton et al. 2010; Wallace 2008) against international bicycling infrastructure design standards for different road types from the Netherlands (CROW 2007) and the United Kingdom (Transport for London 2005). Equations were developed to simulate the effects of the four intervention policies, based on quantitative and qualitative research and local data sources. In particular, we relied on existing systematic (Elvik et al. 2009; Reynolds et al. 2009) and nonsystematic (CROW 2007) reviews of the published and unpublished literature, supplemented by more recently published studies. We considered epidemiological quality and relevance to Auckland to develop point estimates. We also used the lowest and highest confidence limits of all the studies to develop best- and worst-case scenarios and for sensitivity testing. For collision relative risks, we developed region-wide effects based on the proportion of roads treated. The policies are briefly described below, and the effects simulated are summarized in Table 2.

**Table 2:** Effects used in the simulation model for the policy scenarios.

Policy scenario and effects modeled	Studies used	Estimate of effect
RCN: On-road lanes
Risk of collision with a motor vehicle	Elvik et al. 2009; Jensen 2008; Reynolds et al. 2009	0.9
Perception of bicycling safety	Garrard et al. 2008; Jensen et al. 2007; Kingham et al. 2011	4% increase per 10% of network treated
Perception of bicycle commuting	Dill and Carr 2003; Jensen 2008	3% increase per 10% of network treated
RCN: Off-road shared bicycle and foot paths
Risk of collision with a motor vehicle	Aultman-Hall and Hall 1998; Aultman-Hall and Kaltenecker 1999; Elvik et al. 2009	1.0
Perception of bicycling safety	Garrard et al. 2008; Goldsmith 1992	5% increase per 10% of network treated
Perception of bicycle commuting	Aultman-Hall et al. 1997; Buehler and Pucher 2011; Kingham et al. 2011; Larsen 2010	2% increase by doubling km/100,000 population
RCN: Shared bus and bicycle lanes
Risk of collision with a motor vehicle	Newcombe and Wilson 2011	1.0 at mean lane width
ASBL
Risk of collision with a motor vehicle	Elvik et al. 2009; Gårder et al. 1998; Jensen 2008; Lusk et al. 2011; Turner et al. 2011	0.72 midway collisions, 0.8 intersection treatments
Perception of bicycling safety	Garrard et al. 2008; Jensen et al. 2007; Kingham et al. 2011;	6% increase per 10% of network treated
Perception of bicycle commuting	Dill and Carr 2003; Jensen 2008	4% increase every 10% of network treated
SER
Mean local road vehicle speed	Charlton et al. 2010	Proportional increase up to 15 km/hr
Risk of collision with a motor vehicle	Bunn et al. 2003; Charlton et al. 2010; Elvik 2009; Grundy 2009; OECD and European Conference of Ministers of Transport 2006	10-km/hr reduction in speed reduces collisions by 60%
Vehicle volume on local roads	Charlton et al. 2010; Elvik 2009	Proportional increase to 25% reduction
Proportion of bicycle trip distance on local roads	Modest effects based on local expertise	Proportional increase from 50% to 70%
Perception of bicycling safety	Modest effects based on local expertise	Proportional increase up to 10%
Perception of bicycle commuting	Modest effects based on local expertise	Proportional increase up to 10%
Perception of light vehicle convenience	Modest effects based on local expertise	Proportional decrease up to 30%

Regional Cycle Network (RCN). The regional council’s 30-year transport strategy (Auckland Regional Council 2010) includes a commitment to develop a partial network of mixed cycling infrastructure, further details of which were sought directly from the council. Planned infrastructure included on-road marked lanes with no physical segregation on 46% of main roads, an increase from 10 to 25 km of off-road shared footpaths per 100,000 population, and a small number of new shared bus and bicycle lanes (0.04% of the main road network treated). The RCN was simulated to affect cyclists’ risk of collision with a motor vehicle, the proportion of the population considering bicycling to be always or mostly safe, and the proportion of the population considering bicycling to be a good way to get to work.

Arterial segregated bicycle lanes (ASBL). Segregation on Auckland’s arterial roads was assessed to be best practice by mapping Auckland’s arterial roads against the recommendations from the international bicycle design standards. We simulated the effect of gradual implementation of a one-way physically segregated lane on each side of every arterial road in the region by 2051. Studies of physically segregated on-road cycle lanes suggest that without specific intersection treatments, collisions are reduced on midways (between intersections) but may increase at intersections (Elvik et al. 2009; Jensen 2008; Lusk et al. 2011). The most effective intersection treatments reported appear to be transitioning to a marked lane before lighted intersections (Elvik et al. 2009), bicycle stopping areas at the front of traffic lanes (Turner et al. 2011), and elevation across side streets (Gårder et al. 1998). We therefore included these intersection treatments as part of the ASBL policy. Effects on the same variables as the RCN were modeled (risk of collision, perception of safety, and perception of bicycle commuting).

Self-explaining roads (SER). We assessed traffic calming of Auckland’s local roads to be the best practice, using the international design standards. We simulated the gradual transformation of all local through roads into “self-explaining roads” (Theeuwes and Godthelp 1995) by 2050, creating low-speed local streets using nontraditional, endemic road features such as street narrowing, trees, and art (Charlton et al. 2010). We simulated effects of SER on the attractiveness of car use, vehicle speeds, the proportion of bicycle commuting on arterial and local roads, and the perception of both walking and bicycling safety.

Finally, we simulated the effect of a total network best practice policy by combining the ASBL and SER interventions. The costs of each intervention were developed from existing infrastructure costs provided by local and national government stakeholders.

**Figure 2:**
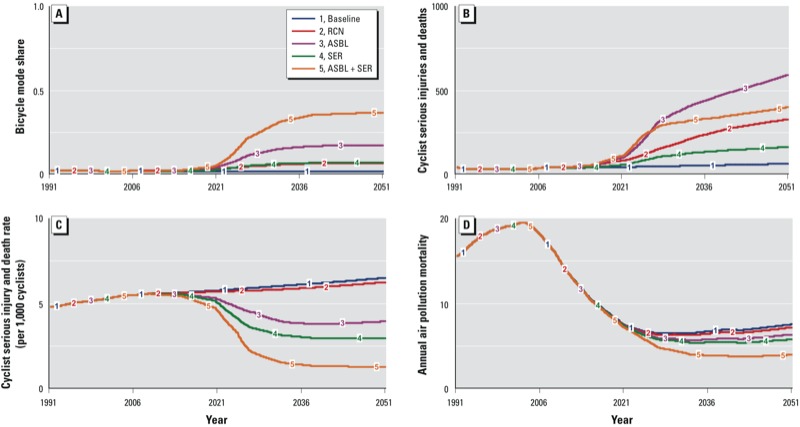
Dynamic model outputs 1991–2051. (*A*) Commuter bicycling mode share. (*B*) Annual serious and fatal injuries to commuter cyclists due to collisions with light vehicles. (*C*) Commuter cyclist injury rate per 1,000 cyclists. (*D*) Mortality due to air pollution from the commuting light vehicle fleet.

Figure 2A shows the simulated effects of each scenario on commuter cycling mode share according to our primary model. Our simulations suggest that all four intervention scenarios would increase the commute mode share of bicycling, up to a mode share comparable to international bicycle-friendly cities, such as Copenhagen, Denmark, and Amsterdam, the Netherlands, both of which have cycle commuting mode shares of about 40% (Copenhagen Technical and Environmental Administration 2013). Auckland has a combined walking and cycling mode share target (35% by 2040) with no indication of how this should be allocated. We have therefore assumed an even split with a mode share target for cycling of 17%. The ASBL and combined best practice policies would be most likely to reach this target by 2040, achieving commute mode shares of approximately 20% and 40% respectively.

The number of bicycling fatal and serious injuries is likely to rise under all scenarios (Figure 2B). The baseline simulation indicates that if cycling remains uncommon, the injury rate for bicycle commuters will increase because of the projected increase in vehicle numbers. The bicycling injury rate is also projected to continue to rise with the RCN. For all other scenarios, the injury rate is projected to fall (Figure 2C). However, an increase in the number of cyclists means that the total number of deaths and serious injuries will rise, most notably in the ASBL scenario. The combined best practice scenario changes the shape of the injury curve (Figure 2B) over time (from sustained growth to plateauing injury numbers) through a rapid increase in bicycling mode share above the threshold required to initiate the safety in numbers feedback.

Reaching Auckland’s strategic target for perception of safety (> 80% of people considering bicycling always or mostly safe by 2040) may be more difficult. The combined ASBL + SER policy is most likely to achieve it based on the simulations, but not until 2051.

Future improved fuel efficiency of the light vehicle fleet means that even without a reduction in the light vehicle mode share, the contribution of the commuting fleet to the region-wide burden of air pollution morbidity and mortality is expected to decline steeply (Figure 2D). Thus, our simulations suggest that the additional air quality benefit of a shift to bicycle commuting would be small. Similarly, greenhouse gas emissions from the commuting fleet are expected to decline slowly under all scenarios (including business as usual), but the combined best practice scenario was the only policy to halve per capita greenhouse gas emissions from the commuting light fleet by 2040 compared with 2007 levels (reducing per capita emissions from approximately 3 metric tons/year to < 1.5 metric tons/year).

All the intervention scenarios achieve savings in all-cause mortality through an increase in population levels of physical activity. The savings range from tens of lives per year (RCN and SER) to hundreds of lives per year (ASBL and combined best practice).

There are projected fuel cost savings under all scenarios (including slight savings even with no intervention), which will increase over time if rising fuel prices outstrip improvements in light vehicle fuel efficiency. According to our simulations, the RCN and SER scenarios would achieve annual fuel cost savings in hundreds of millions of $NZ due to increased commuter bicycling, while the ASBL and combined best practice scenarios would achieve annual savings in billions of dollars (Table 3).

**Table 3:** Cumulative outcomes projected from the simulation of active policy scenarios compared with the business-as-usual scenario.

Outcome	RCN (monetized)	ASBL (monetized)	SER (monetized)	ASBL + SER (monetized)
Cycling mode share by 2051 (%)	5	20	5	40
LV mode share by 2051 (%)	75	65	55	40
Proportion of people considering cycling always/mostly safe by 2040	0.4	0.7	0.3	0.9
LVKT (billion km)	–3.5	–7	–10	–18.5
Cyclist injuries
Fatalities	200 (620)	360 (1,100)	85 (250)	250 (850)
Serious injuries	4,000 (1,300)	7,000 (2,300)	1,600 (500)	5,000 (1,600)
Car crashes
Car occupant fatalities	–70 (–220)	–120 (–370)	–170 (–527)	–340 (–1,000)
Air pollution
Mortality	–10 (–7.5)	–20 (–15)	–40 (–30)	–80 (–60)
Hospitalizations	–5 (–0.02)	–15 (–0.04)	–20 (–0.06)	–40 (–0.12)
COPD incidence	–10 (–0.75)	–30 (–2.25)	–55 (–4)	–90 (–6.75)
Restricted activity days	–12,500 (–1)	–37,200 (–4)	–57,700 (–6)	–112,200 (–11)
Air pollution total	(–9)	(–21)	(–40)	(–78)
All-cause mortality	–650 (–2,000)	–1,850 (–5,700)	–650 (–2,000)	–4,000 (–12,400)
Greenhouse gas emissions (megatons)	–3 (–120)	–8 (–360)	–13 (–520)	–26 (–1040)
Fuel cost ($NZ million)	(–600)	(–1,800)	(–600)	(–3,900)
Infrastructure cost ($NZ million)	(45)	(250)	(380)	(630)
Net benefit ($NZ million)	–770	–2,550	–1,780	–13,090
Benefit–cost ratio	18	18	6	24
Abbreviations: LV, light vehicle; LVKT, light vehicle kilometers travelled. Numbers with a negative sign represent ­savings. Monetized figures are given in parentheses and are in millions of New Zealand dollars. Benefit–cost ratios are calculated from the net public health benefits and infrastructure costs shown.				

Table 3 illustrates the estimated cumulative costs and benefits to 2051 of the four policy interventions compared with the business-as-usual simulation. All interventions exhibit positive benefit–cost ratios, ranging from $NZ6 to > $NZ20 saved for every dollar spent on bicycling infrastructure. The largest savings come from reductions in all-cause mortality due to physical inactivity. However, the distribution and magnitude of costs and savings vary widely between scenarios. Although the RCN is cheap to build (~ $NZ45 million), it achieves only a small shift in mode share accompanied by a large injury burden, resulting in net benefits in the hundreds of millions of dollars. Yet the combined best practice scenario, although considerably more expensive to build (approximately $NZ630 million), achieves a much larger mode shift with a similar number of bicycling injuries and a cumulative net benefit in tens of billions of dollars, as shown in Table 3. In this scenario, bicycling fatalities are offset by the reduction in other motor vehicle injuries. Because a major effect of the SER intervention is to reduce vehicle use by making it less convenient (Charlton et al. 2010), rather than to directly increase bicycling, further effects of the SER scenario will accrue through increases in all noncar transport modes (including walking and public transport). Therefore, counting only the benefits and costs due to an increase in commuter bicycling reflects only part of the benefits and costs of this intervention.

*Policy sensitivity analysis.* The results of the policy parameter testing are summarized in the Supplemental Material, Tables S4–S7 and Figures S1–S6. Realistic changes to policy assumptions did not change the shape of outcome behaviors over time. However, injury outcomes were sensitive to safety-in-numbers assumptions. In particular, simulating the no-threshold power function proposed by Jacobsen (2003) altered the shape of change over time of injury outcomes for all scenarios, but worsened the validity against historical data.

The Monte Carlo analysis demonstrated order-of-magnitude variability in mode share outcomes only for the SER policy. Overlap in the mode share effects of some policies was evident, but the model continued to distinguish among the RCN, ASBL, and combined best practice policies (see Supplemental Material, Table S7 and Figure S6). Simulating the reported range of effects of commuter cycling on all-cause mortality altered the order of magnitude of savings, and the model was not able to distinguish between scenarios because of overlapping effects. Plausible variation in lead times for physical activity benefits led to order-of-magnitude differences in outcomes, while retaining the ability to distinguish between the RCN and combined best practice scenarios.

Even under a best-case scenario, the RCN would result in an increase in the cyclist injury rate (Figure 2C; see also Supplemental Material, Figure S1) because the infrastructure components have the potential to make cycling more dangerous, while failing to achieve the mode shift necessary to reduce vehicle growth or reach the safety in numbers threshold. On the other hand, policies 4 (SER) and 5 (ASBL + SER) reduce the cycling injury rate even under worst-case scenario testing.

## Discussion

*Principal findings*. Under our primary model assumptions, the benefits of all the intervention policies outweighed the harms, between 6 and 24 times. However, there were order-of-magnitude differences in estimated net benefits among policies. A universal approach to bicycle-friendly infrastructure will likely be required to achieve sufficient growth in bicycle commuting to meet strategic goals. Our findings suggest that the most effective approach would involve physical segregation on arterial roads (with intersection treatments) and low speed, bicycle-friendly local streets. We estimate that these changes would bring large benefits to public health over the coming decades, in the tens of dollars for every dollar spent on infrastructure. The greatest benefits accrue from reduced all-cause mortality due to population-level physical inactivity.

The modeling enabled discussions about the forces behind bicycling trends between policy and nongovernment actors, based on a shared structural understanding. By developing a more consistent, shared, structure-based understanding, we expect this participatory modeling process to reduce conflict among policy stakeholders and successfully support policy change. Simulating the model delivered further insights, identifying feedback loops that were inactive and enabling comparison between plausible policies to demonstrate how strategic targets could be met. Conflicting, mismatched, and unrealistic targets were also identified. For example, meeting the postulated bicycling mode share target was possible through several of the policies simulated. However, none met the perception of safety target. Except for the combined best practice policy, increases in cycling mode share conflicted with targets to reduce road traffic deaths and injuries. The dynamic output graphs suggest that the planned RCN is unlikely to meet regional targets and fails to address the projected business-as-usual increase in the bicycling injury rate. Through the integrated modeling of benefits we were able to show that a more ambitious approach would be cost-effective.

*Strengths and weaknesses*. To our knowledge, this is the first integrated assessment of specific alternative active transport policies, and one of the first uses of SDM to integrate health, social, and environmental outcomes of urban policies.

The study builds on previous integrated assessments of transport policies (e.g., Lindsay et al. 2011; Rojas-Rueda et al. 2011; Woodcock et al. 2009). The model improves our understanding of active transport policy impacts by circumventing the limitations of both small-scale, rigorous interventions (Ogilvie et al. 2004) and larger ecological comparisons (Buehler and Pucher 2011). By combining local stakeholder knowledge, regional data, and epidemiological evidence using SDM, we were able to combine the structural and the social influences on cycling uptake with evidence-based and context-specific emphasis given to each.

The method allowed us to model bicycling collisions and injuries with greater sophistication, including a more nuanced approach to safety in numbers and a more sophisticated model of the effects of traffic speed than previous studies. It also enabled us to account for expected dynamic changes in all-cause mortality and the light vehicle fleet, and to undertake a deeper analysis of structural and parametric uncertainty. However, we were able to include only bicycling injuries occurring as a result of a collision with a light vehicle. Collisions with heavy vehicles, other cyclists, and pedestrians and cyclist-only injuries were not included. Although collisions with motor vehicles account for > 90% of the serious injuries and fatalities reported in New Zealand’s crash analysis, other kinds of cyclist injuries are also likely to increase with increasing bicycle mode share.

The relative risks from bicycling cohort studies used in our model may overestimate the mortality benefit of commuter bicycling. These relative risks are larger than expected from meta-analyses of physical activity (Löllgen et al. 2009; Woodcock et al. 2011). Conversely, there are likely benefits not yet able to be counted because of a lack of quantified evidence. These include reductions in inactivity-related morbidity, increased social connection, and local economic benefits.

The development of a useful SDM requires a balance between parsimony and accuracy. Consistent with most SDM endeavors, our goal was to develop a reflective model for enhancing communication and understanding of system structure and behavior. Although the assumptions meant it was not possible to make point predictions of policy effects, robust policy insights can to be drawn despite mixed quality data.

*Implications for policy and practice*. In high-income, car-dependent cities such as Auckland, particular bicycle-friendly interventions will be crucial to turn patterns of declining commuter cycling into sustained growth that would meet climate and health goals. Although our findings suggest that Auckland’s existing plan to develop a regional cycle network would likely have benefits, the simulation modeling suggests that it would not reverse the predicted business-as-usual increased rate of cycling injury. In contrast, a gradual transformation of all roads using best practice arterial and local street interventions could make a major contribution to regional transport targets. Our projections suggest that, assuming our assumptions are valid, this transition would be cost-effective, returning tens of dollars in benefits for every dollar spent.

Participatory SDM represents a methodological step forward for health impact assessment. As well as being applicable to other transport and land use policies, it could assist with intervening in the wider interdependent systems driving obesity, physical inactivity, and climate change.

*Implications for future research*. The sensitivity analysis identified important assumptions, and therefore future research directions. The model was most sensitive to assumptions about safety in numbers, highlighting the need for longitudinal studies of cycling injuries accounting for both infrastructure and cyclist numbers.

Although the causal loop diagram is context specific, we find it a useful basis for discussions in other cities. Further qualitative research is needed to assess the generalizability of the feedbacks identified in this study. The model equations and data sources may also act as a template for similar research elsewhere.

Extending the approach taken in this study could identify intervention patterns that reduce health and social inequalities. Further research is also needed on benefits and costs that have not been counted to date, enabling a more complete assessment of active transport policies. These include other possible positive effects of a shift from cars to bicycles, such as improvements in water quality, improved workplace productivity, reduced morbidity associated with physical inactivity, and financial savings from reduced demand for new roads and urban car parks as well as effects on other kinds of bicycle trips than commuting. In addition, other negative effects are also possible, such as bicycling injuries other than those resulting from a collision with a motor vehicle.

## Supplemental Material

(1.5 MB) PDF
