# Supplementary material for: The Societal Costs and Benefits of Commuter Bicycling: Simulating the Effects of Specific Policies Using System Dynamics Modeling
Source: Environ Health Perspect. 2014 Feb 4;122(4):335–44. doi: 10.1289/ehp.1307250 (PMC3984216; doi:10.1289/ehp.1307250)
Supplement: (1.5 MB) PDF [file ehp.1307250.s001.pdf]

Environmental Health Perspectives doi:10.1289/ehp.1307250

Supplemental Material

The Societal Costs and Benefits of Commuter Bicycling: Simulating the Effects of Specific Policies Using System Dynamics Modeling

Alexandra Macmillan, Jennie Connor, Karen Witten, Robin Kearns, David Rees, and Alistair Woodward

Table of Contents **Page**

Summary of stakeholders **3**

Table S1. Groups represented in the participatory system dynamics modelling process. 3

Sensitivity analysis **4**

Table S2. Summary of formal validation procedures. 4

Table S3. Parameters tested and approach used in the sensitivity analysis of the simulation model. 5

Policy parameter sensitivity testing **9**

Infrastructure costs *9*

Table S4. Estimated costs per km of policy interventions. 9

Table S5. Sensitivity ranges for total and average annual costs of intervention policies (million NZ dollars). 9

Regional cycle network (RCN) *10*

Figure S1. Upper and lower bounds for cycling injury outcomes under extremes for RCN component relative risk of cycle-vehicle collision. 11

Arterial segregated cycle lanes (ASBL) *12*

Figure S2. Upper and lower bounds for cycling injury outcomes under extremes for ASBL relative risk of cycle-vehicle collision. 13

Self explaining local roads (SER) *14*

Table S6. Sensitivity ranges for all mode shares under the self explaining roads policy. 14

Mixed universal policy (ASBL + SER) *15*

Figure S3. Upper and lower bounds for cycling injury outcomes under extremes for ASBL + SER relative risks of cycle-vehicle collision and the proportion of cyclists travelling on arterial roads. 15

Figure S4. Range of injury outcomes for ASBL + SER policy seen under random simulation across normal distributions for the effect of components on collisions and the proportion of cyclists travelling on arterial roads. 16

Figure S5. Mode share outcomes for ASBL + SER policy simulated using random sampling from normal and uniform distributions of component criteria. 17

Monte Carlo analysis *18*
Table S7. Range of mode share and annual injury outcomes for all scenarios from the sensitivity analysis of policy assumptions. 18
Figure S6. Mode share outcomes for all scenarios from the Monte Carlo sensitivity analysis. 18

Simulation model equations **20**
Commuting patterns *20*
Mode share *21*
Commuting vehicle kilometres travelled *26*
Commuter cyclist injury *27*
Air pollution outcomes *30*
Physical activity related mortality *33*
Fuel costs *37*
Greenhouse gas emissions *38*
RLTS 2010 Regional Cycle Network *39*
ASBL Policy *40*
SER Policy *41*
Intervention costs *42*

References **45**

Summary of stakeholders

Table S1 summarises the stakeholders involved in the participatory system dynamics modelling, including the interviews and workshops to develop the qualitative model. In addition, a Māori steering group included 15 regional representatives. There was overlap in representation between the groups as some stakeholders represented more than one of the target groups. The groups represented were based on the requirements of the NZ Land Transport Management Act ([Anonymous] 2003).

Table S1. Groups represented in the participatory system dynamics modelling process.

Groups represented	Number of participants
People with disabilities	1
Māori communities	5
Pacific communities	3
Low income families	3
Young people	2
Regional transport policy makers	2
National transport agency	1
Public health	2
Local business association	1
Local tertiary institution	2
Local government	2
Regional government	2
Academics	3

People with disabilities self-identified as such and were represented by a member of the local council’s disabilities steering group. Maori and Pacific representatives identified themselves as belonging to these ethnic groups and were drawn from a network of governmental and non-governmental organisations. Young people were defined as aged younger than 18. Public health representatives included professionals working at the Auckland Regional Public Health Service and public health academics.

Sensitivity analysis

Table S2. Summary of formal validation procedures.

Formal validation	Specific procedures	Description
Direct structure validity	Parameter confirmation	Consistency with elements of the real system; Numerical accuracy of constants; Parameters chosen from best evidence.
Direct structure validity	Structure confirmation	Equations reflect real relationships, conforming to physical laws; Relationships based on best evidence; Extreme conditions testing of relationships; Dimensional consistency testing
Structure-oriented behaviour	Extreme-condition procedures	Plausible behaviour with extreme values
Structure-oriented behaviour	Behaviour sensitivity procedures	Identification of parameters to which the model is highly sensitive
Behaviour pattern validity	Pattern consistency for transient behaviours	Graphical and visual comparisons with historical time series data

Adapted from Forrester and Senge (1980), Barlas (1996), Sterman (2000) and van den Belt (2004).

Table S3. Parameters tested and approach used in the sensitivity analysis of the simulation model.

Parameter	Comments on data	Approach to sensitivity analysis
<u>**Mode share and population**</u>		
Change in commuting population	Census data trends used in forecast modelling	Normal distribution, upper and lower bounds for population growth (1 and 1.8%)
Mode normal	No data available – stakeholder opinion	Uniform distribution 0.5-1.0
Time to change behaviour	Judgement	Random distribution 2 months-3 years
<u>**Outcomes**</u>		
**Air pollution**		
PM_{10} per km effects	Results of observational studies combined with geographical modelling of vehicle pollution used to develop per km travelled effects specific to Auckland (HAPiNZ 2010)	Considered underestimate, per km effects doubled, normal distribution with mean 1.5 times best estimate and standard deviation best estimate/6
Light vehicle PM_{10} emission improvements	Fleet measurements	Level of uncertainty not known – no further improvement and double improvement tested
**Injury**		
Ratio of commuting light vehicles to other light vehicles at peak time	No data available – stakeholder opinion	Uniform distribution 0.4-0.8
Ratio of light vehicles arterial:local roads at peak times	No data available – stakeholder opinion	Uniform distribution 0.7-0.9
Ratio of cyclists arterial:local roads at peak time	No data available – stakeholder opinion	Uniform distribution 0.3-0.7
Safety in numbers	Ecological studies, administrative injury and census data, stakeholder opinion	Sensitivity to Jacobsen's assumption and and a lower threshold (5% suggested by Turner)
Effect of vehicle numbers on collisions	Crash prediction modelling based on crash, cycle count and motor vehicle count data for New Zealand cities	Know there is a 0,0 point and current motor vehicle and crash counts, but sensitivity tested to altering the shape of the graphical function
Effect of LV speed on crash fatality ratio	Accident reconstruction, mathematical modelling,	Difficult to do – from injury data and speed data we can work out where we are on the graph, and the relationship between speed and CFR is reasonably well accepted – not tested

Parameter	Comments on data	Approach to sensitivity analysis
**Physical activity**		
Effect of cycling on all-cause mortality	Cohort studies of commuter cycling – assume a linear dose response, and that "average" commuter cycling can be representative	Normal distribution using SDs from cohort studies (RR 0.57-1.01): mean 0.79, standard deviation 0.07
Lead-in/lead-out times for effect of physical activity on all-cause mortality	Implicit in cohort studies, interpreted by the candidate	Uniform distribution between 1 and 5 years
**Greenhouse gas emissions**		
LV fleet CO_2 emissions	VEPM model	Uncertainty not known, but likely to grow with time - no further improvement in LV CO_2 emissions and double the improvement projected by VEPM tested
**Fuel cost savings**		
Fuel consumption	VEPM model	Uncertainty not known, but likely to grow with time - no further improvement in LV fuel consumption and double the improvement projected by VEPM tested
Diesel and petrol costs	Forecast modelling combining expectations of extraction and refining on the supply side, with growing market demand	Normal distribution, using upper and lower bounds from Donovan, et al.
**Policies**		
Infrastructure costs	Auckland Transport estimates	Normal distribution: means mid-point of range provided, standard deviations calculated as the range/6
**Regional cycle network**		
Effect of on-road lanes on collisions	Poorly controlled before-after studies	Wide confidence intervals in studies tested using normal distribution of relative risk (0.83-1.5)
Effect of on-road lanes on sense of safety	Stated preference survey of cyclists in Copenhagen	
No confidence intervals reported but comparative differences supported by other studies	Normal distribution, mean 0.58, standard deviation 0.06	
Effect of on-road lanes on cycling good for work	No data available, candidate judgement supported by stakeholder opinion	Uniform distribution between 0 and 0.5

Parameter	Comments on data	Approach to sensitivity analysis
Effect of off-road tracks on collisions	Poorly controlled before-after studies	Wide confidence intervals in studies tested using normal distribution (0.9-3.5)
Effect of off-road tracks on sense of safety	Ecological study with dose-response combined with judgement	Normal distribution mean 0.4, standard deviation 0.06
Effect of off-road tracks on cycling good for work		Uniform distribution 0 to 0.3
Effect of bus-bike lanes on collisions	Poorly controlled before-after study	Confidence intervals not supplied. Range of point estimates in study used
Effect of bus-bike lanes on sense of safety	Judgement supported by stakeholder opinion	Uniform distribution 0 to 0.3
Effect of bus-bike lanes on cycling good for work	Judgement supported by stakeholder opinion	Uniform distribution 0 to 0.4
**Arterial separated bike lanes (ASBL) and best practice intersections (BPI)**		
Effect of ASBL on collisions	Controlled before-after studies	Normal distribution of relative risk using upper and lower bounds from all studies (0.6-1.1)
Effect of ASBL on sense of safety	Stated preference survey of cyclists in Copenhagen	
No confidence intervals reported but comparative differences supported by other studies	Normal distribution of effect with mean 0.6 and standard deviation 0.06	
Effect of ASBL on good for work	Judgement supported stakeholder opinion	Uniform distribution of effect 0.2-0.6
Effect of BPI on collisions	Poorly controlled before-after studies	Normal distribution of relative risk using upper and lower bounds from studies of advanced stop lines (0.61-1.16)
Effect of BPI on sense of safety	Judgement supported by stated preference survey in Copenhagen	Uniform distribution of effect between 0 and 0.2
**Self explaining roads (SER)**		
Effect of SER on collisions	OECD speed management report – source data not known	
Estimate taken as best case scenario	Uniform distribution of relative risk between 0.4 and 0.8	
Effect of SER on cyc good for work	Judgement supported by stakeholder opinion	Uniform distribution between 0 and 0.3

Parameter	Comments on data	Approach to sensitivity analysis
SER on cycling sense of safety	Judgement supported by stakeholder opinion	Uniform distribution between 0 and 0.4
SER on light vehicles hassle free	Combined reduction and diversion of traffic supported by local and international before-after studies	Normal distribution, mean -0.3, standard deviation 0.06
SER on LV arterial		Normal distribution between 0.55 and 0.95
SER on proportion cycling arterial	Judgement supported by stakeholder opinion	Uniform distribution between 0.3 and 0.9
SER local speed	Local controlled before-after study supported by international studies	Normal distribution 20-40kph

Table S3 describes the variables tested, including the distributions used. Decisions to include variables in the sensitivity analysis were made on the basis of multi-dimensional considerations, including aspects of study design, data completeness, applicability to the Auckland context, and how well the data fit the model purpose. Judgement about multi-dimensional aspects of data quality was used to identify the most uncertain data sources that are also most likely to affect the model behaviourally. For data from cohort studies, modelled forecasts using sampled measurements and controlled before-and-after studies I have used reported confidence intervals as the range for sensitivity testing and a normal distribution for Monte Carlo simulation. For survey data I have used judgement to identify a range and a normal distribution for the Monte Carlo simulation. For variables based on expert opinion I have used judgement to identify a wider range and a uniform distribution for the Monte Carlo simulation. For parameters with a normal distribution, standard deviations were calculated such that 3 standard deviations included upper and lower bounds.

Policy parameter sensitivity testing

Infrastructure costs

The sensitivity of total and annual policy costs was tested to the range of infrastructure costs provided by Auckland transport (summarised in Table S4). The best estimate cost for the SER pilot study was used as an upper bound (range $100,000-300,000). A range of $100,000-300,000 was also used for the ASBL policy.

The ranges for total and annual average costs to 2051 under all policy scenarios are shown in Table S5. The model is order-of-magnitude sensitive to these assumptions for scenarios 2 and 5.

Table S4. Estimated costs per km of policy interventions.

Intervention	Cost per km range (best estimate)	Cost per km modelled
On-road marked cycle lane (2 sides)	$5-40,000 ($15,000)	$15,000
Shared bus-bike lane (2 sides)	$50-200,000 ($100,000)	$100,000
Off-road shared path	$75-400,000 ($150,000)	$150,000
On-road separated lane + intersection treatment	None supplied	$200,000
Self explaining local road	$300,000	$300,000

Table S5. Sensitivity ranges for total and average annual costs of intervention policies (million NZ dollars).

Scenario	Total cost to 2051 (million NZ dollars)	Average annual cost (million NZ dollars)
2 (RCN)	128-715	4.4-24.7
3 (ASBL)	754-2262	19.3-58
4 (SER)	759-2278	19.5-58
5 (ASBL + SER)	1513-4540	39-116

Regional cycle network (RCN)

A best and worst case scenario for each uncertain parameter was undertaken. For the effect on collision, differences in mode share, perception of safety and injury outcomes are described. For other variables only the varying effect on cycle mode share is reported.

Worst and best case scenarios were simulated using the range of reported confidence intervals for component relative risks of cycle crashes in the literature. A best case scenario for the effects on the RCN on cyclist-vehicle collisions sees the RCN result in a cycling commute mode share of nearly 6% by 2051, with 40% people considering cycling always/mostly safe. By 2051 there would be 310 fatal and serious injuries/year, and a serious injury rate of 6/1000 cyclists per year and gradually rising because of growing vehicle numbers. In a worst case scenario, the same mode share is achieved, with a similar perception of safety, but 500 fatal and serious cyclist injuries and a large increase in the rate of serious injury to 10/1000 cyclists per year. The behavioural stability of injury outcomes under these upper and lower bounds is demonstrated in Figure S1.

Running 30 simulations that randomly sampled from normal distributions for the component relative risks across the confidence intervals indicated by the literature gives a narrower and more conservative range for the injury outcomes: 200-335 serious and fatal injuries per year by 2051 with an injury rate of between 6.3 and 8.9/100,000 cyclists per year.

Random simulation from normal and uniform distributions for the effects of aspects of the RCN on sense of safety results in a range of cycling commute mode shares under the RCN of 4 to 7%. Random simulation across a uniform distribution for the effects of RCN components on cycling as a good mode for work results in a range of effects on cycling commute mode share between 2 and 8% by 2051.

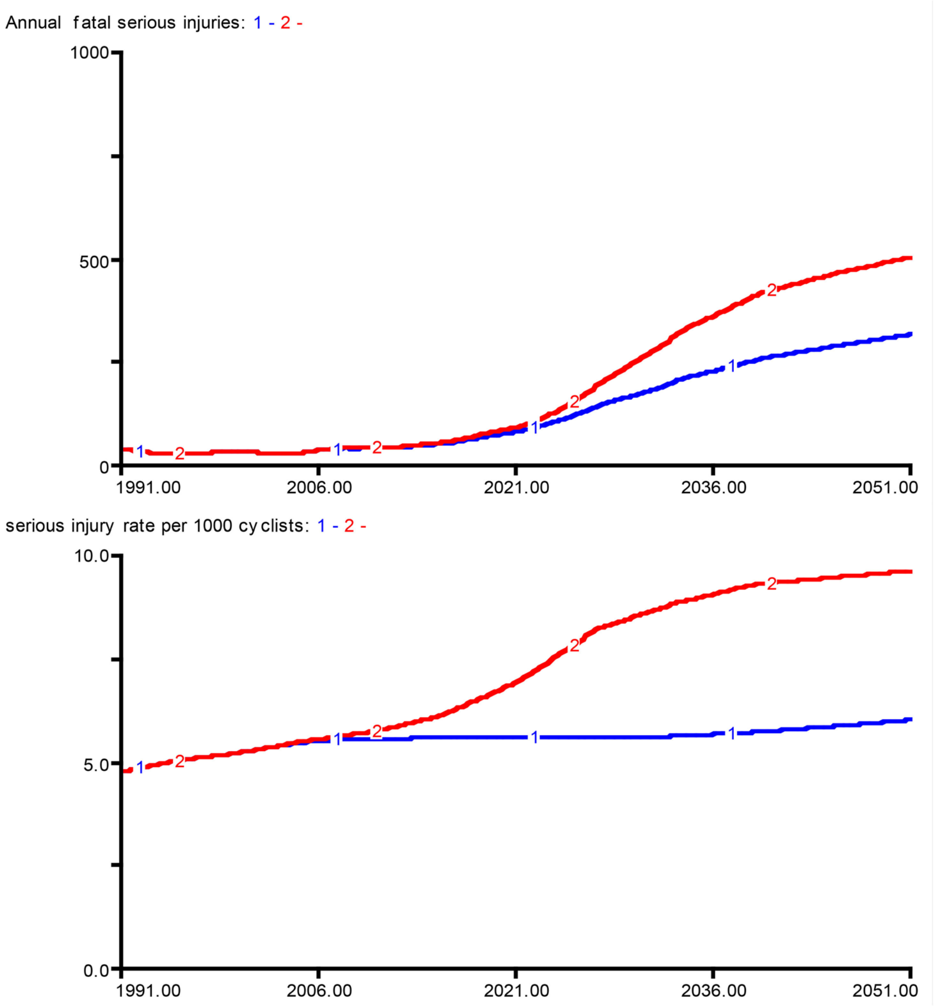

Figure S1. Upper and lower bounds for cycling injury outcomes under extremes for RCN component relative risk of cycle-vehicle collision.

Arterial segregated cycle lanes (ASBL)

Using upper and lower bounds from confidence intervals reported in the literature, a best case scenario for the combined effect of ASBL with best practice intersections on cyclist-vehicle collisions sees a cycling commute mode share of 18% by 2051 with 70% people considering cycling always/mostly safe. Five hundred fatal/serious injuries per year result by 2050 (similar to the worst case scenario for the RCN) but with a serious injury rate of 3.3/1000 cyclists per year. The worst case scenario has a similar effect on mode share and perception of safety but with 960 fatal/serious cyclist injuries per year and an injury rate of 6.4/1000 cyclists per year, slightly increasing over time. The behaviours of cycling injury outcomes over time for the best and worst cases are shown in Figure S2.

Analysing across 30 simulations using a normal distribution for these two relative risks again provides a narrower range of 233-563 serious and fatal injuries/year by 2051 and a serious injury rate of 3.57-5.75/1000 cyclists/year.

Random simulation across appropriate normal and uniform distributions for effects of the ASBL policy on cycling sense of safety and cycling good for work results in cycling mode shares between 10 and 24% by 2051.

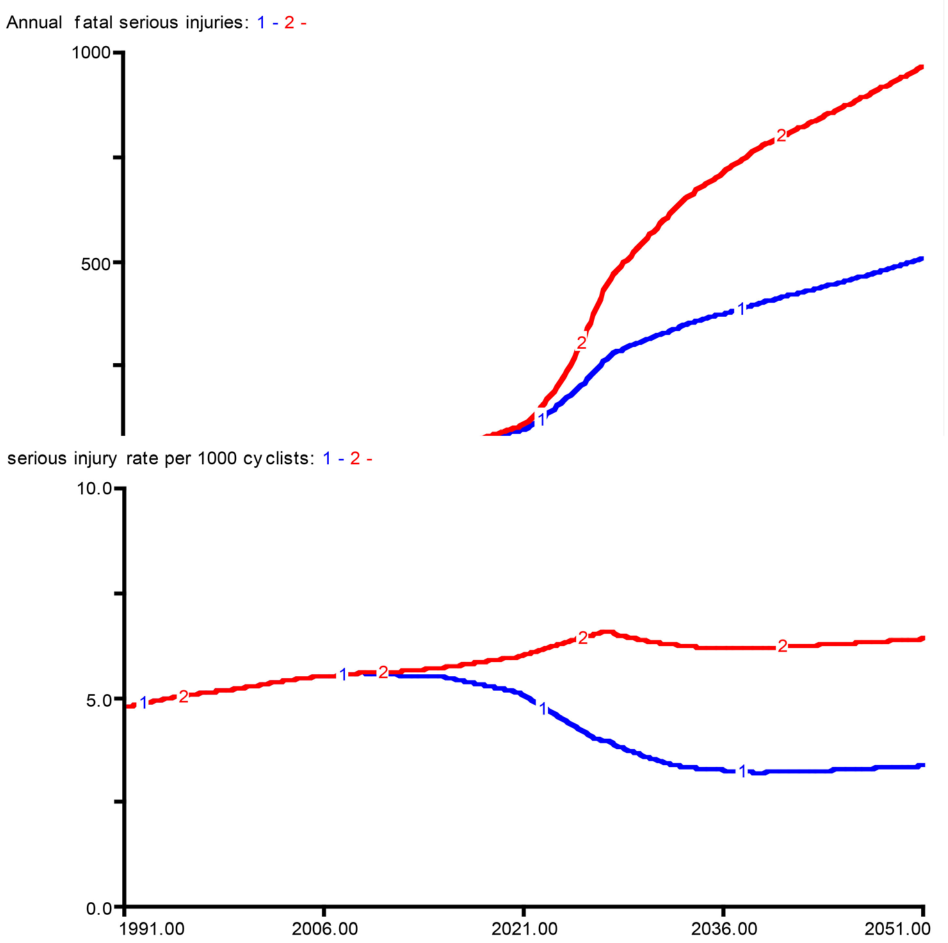

Figure S2. Upper and lower bounds for cycling injury outcomes under extremes for ASBL relative risk of cycle-vehicle collision.

Self explaining local roads (SER)

Worst and best case scenarios for the effect of SER on vehicle-cyclist collisions have similar effects on mode share. In a best case scenario this policy results in 158 serious/fatal cyclist injuries per year by 2051, and an injury rate of 2.9/1000 cyclists/year. At its worst the SER policy has similar effects, resulting in 182 serious/fatal injuries per year and an injury rate of 3.4/1000 cyclists per year by 2051.

Other effects of SER policy also have an impact on cycling injury outcomes. Best and worst case estimates for the effect of SER on the average speed of local roads result in a narrow range of annual serious/fatal cyclist injuries between 145-180 and a range of injury rates between 2.6 and 3.3/1000 cyclists per year. Testing the effect of SER on the proportion of peak time light vehicles on arterial and local roads using a range of reductions between 5 and 45% makes no difference to injury outcomes. Injury outcomes for this policy are most sensitive to assumptions about the effect of SER on the proportion of peak time cycling spent on arterial roads (baseline is 50%). A range of effects between a reduction to 45% and a reduction to 15% results in a range of serious/fatal injuries of 111-212/year and a range of injury rates between 1.8 and 4/1000 cyclists by 2051.

Random simulation across normal and uniform distributions for the effect of the SER policy on cycling perception of safety, cycling good for work and light vehicles hassle free results in the wide ranges for mode shares seen in Table S6.

Table S6. Sensitivity ranges for all mode shares under the self explaining roads policy.

Mode share	Range
Cycling	0.02-0.16
Light vehicle	0.4-0.7
Public transport	0.16-0.37
Walking	0.08-0.18

Mixed universal policy (ASBL + SER)

Best and worst case scenarios were tested using the range of collision rates for the components of policies tested earlier, as well as the range for the SER proportion of cyclists on arterial roads. These two simulations result in a range of serious and fatal injuries between 223 and 1177/year by 2051 and injury rates between 0.7 and 3.6/1000 cyclists per year (Figure S3).

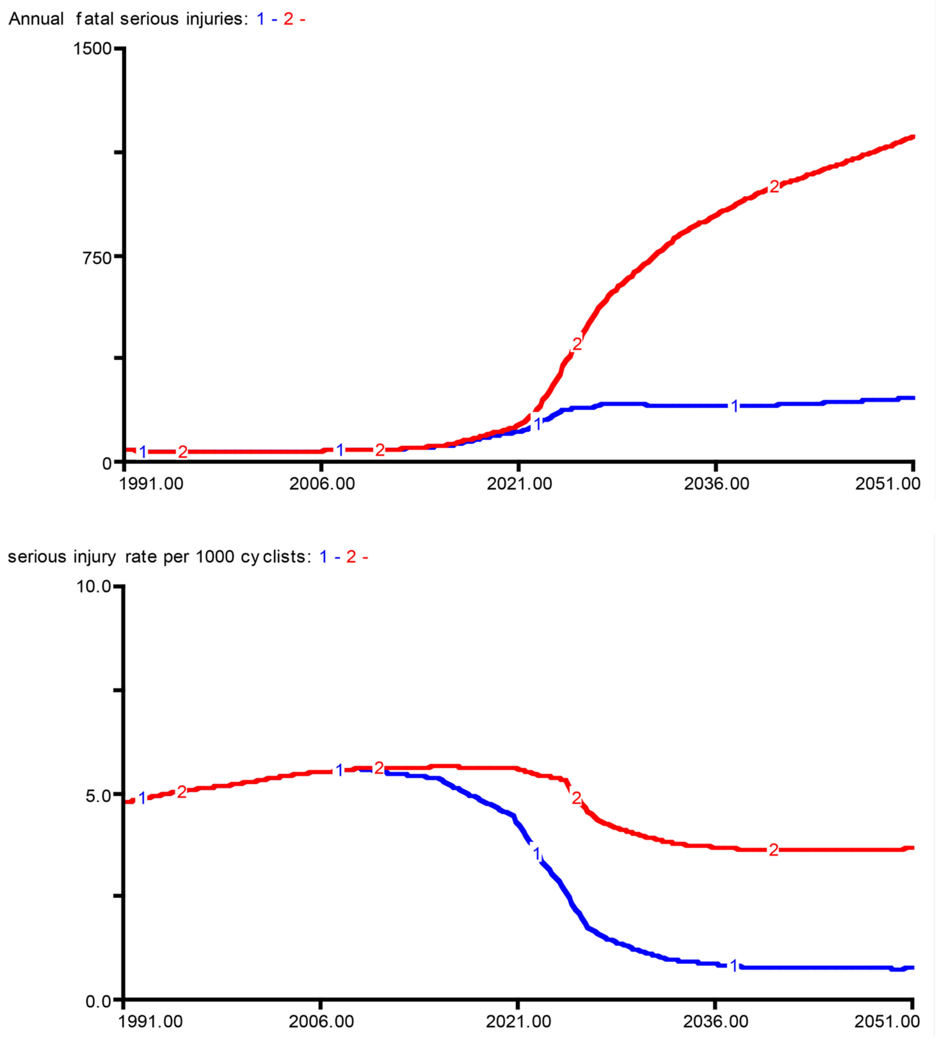

Figure S3. Upper and lower bounds for cycling injury outcomes under extremes for ASBL + SER relative risks of cycle-vehicle collision and the proportion of cyclists travelling on arterial roads.

Simulating this policy over a range of effects using normal distributions for the relative risk estimates results in a narrower range of cyclist serious fatal injuries (201-755 per year by 2051) and injury rates (1.1 to 2.7/1000 cyclists per year by 2051), as shown in Figure S4.

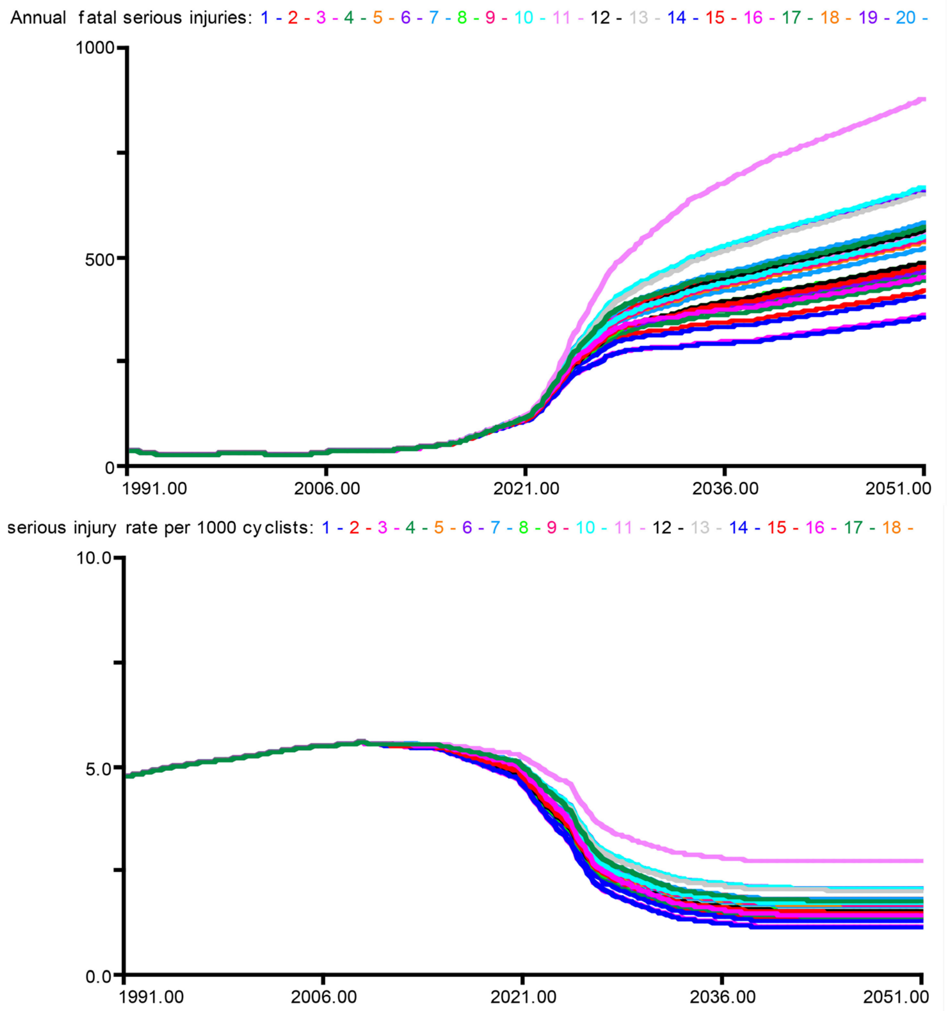

Figure S4. Range of injury outcomes for ASBL + SER policy seen under random simulation across normal distributions for the effect of components on collisions and the proportion of cyclists travelling on arterial roads.

Multiple simulations combining the normal and uniform distributions for the effects of all the components of this scenario on the determinants of mode share result in a behaviour and order-of-magnitude stable range of cycling mode share between 20 and 52% by 2051 (Figure S5).

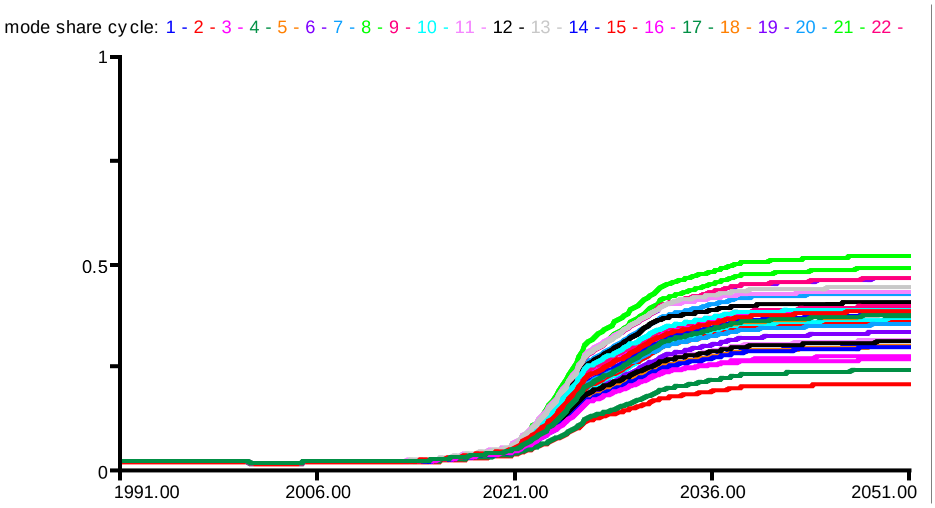

Figure S5. Mode share outcomes for ASBL + SER policy simulated using random sampling from normal and uniform distributions of component criteria.

Monte Carlo analysis

A Monte Carlo approach was used to randomly sample from distributions of variables determining mode share to provide a range for the mode share outcomes of each scenario. The results of these analyses are summarised in Table S7.

Table S7. Range of mode share and annual injury outcomes for all scenarios from the sensitivity analysis of policy assumptions.

Outcome	Scenario 2 (RCN)	Scenario 3 (ASBL)	Scenario 4 (SER)	Scenario 5 (ASBL + SER)
**Cycling mode share**	0.02-0.08	0.1-0.24	0.02-0.16	0.2-0.52
**Annual serious and fatal injuries**				
Worst and best case scenarios	310-500	500-960	111-212	223-1177
Monte Carlo analysis	200-335	233-563	N/A	201-755

It can be seen that cycling mode share is order-of-magnitude sensitive to assumptions under scenarios 3 and 4. Some overlap between scenarios is also evident from the Monte Carlo analysis (Figure), but the model retains its ability to distinguish between scenarios 2, 3 and 5.

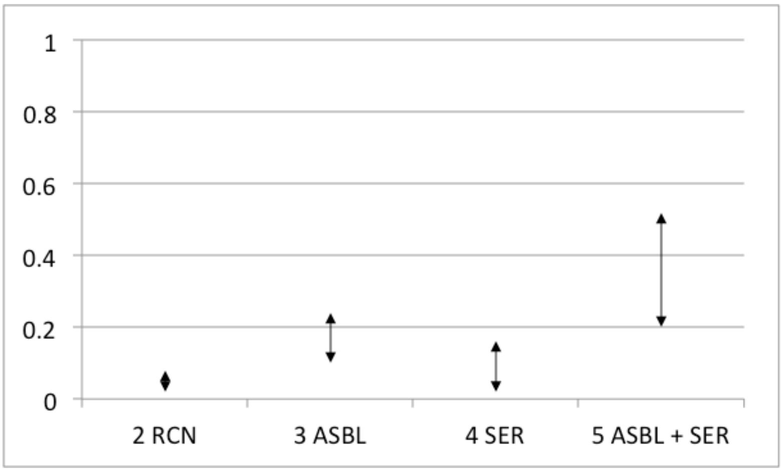

Figure S6. Mode share outcomes for all scenarios from the Monte Carlo sensitivity analysis.

Annual injury outcomes exhibit less overlap than the mode share outcomes overall, although a greater degree of overlap was again seen in the injury ranges for scenarios 3 and 5.

Assumptions about safety in numbers and variables influencing the effect of commuter cycling on all-cause mortality were tested separately. Changing the threshold for the safety in numbers effect to 5% cycling mode share did not alter the behaviour or order of magnitude of injury outcomes. However, simulating the power function from Jacobsen with no threshold changed the behaviour of injury outcomes. Simulating the range of relative risks of all-cause mortality for commuter cycling (using a Monte Carlo approach with the confidence intervals in the literature) altered the order of magnitude of all-cause mortality savings for all scenarios and disabled the ability of the model to distinguish between any of the active interventions. Monte Carlo simulation of a plausible range of lead times for physical activity benefits to accrue led to order-of-magnitude differences for scenarios 2 and 5, while only retaining the ability of the model to distinguish between scenario 2 and 5.

Simulation model equations

Commuting patterns

cycle commuters(t) = Cycle commuters(t - dt) + (change in cycling) * dt

INIT Cycle commuters = Commuter cyclists initial

INFLOWS:

change in cycling = ((Commuters*mode share cycle)-Cycle commuters)/time to change behaviour

light Vehicle Commuters(t) = Light Vehicle Commuters(t - dt) + (change in LV use) * dt

INIT Light Vehicle Commuters = Light vehicle Commuters   initial

INFLOWS:

change in LV use = ((Commuters*mode share LV)-Light Vehicle Commuters)/time to change behaviour

public transit commuters(t) = public transit commuters(t - dt) + (change in PT use) * dt

INIT public transit commuters = public transport initial

INFLOWS:

change in PT use = ((Commuters*mode share PT)-public transit commuters)/time to change behaviour

walking commuters(t) = walking commuters(t - dt) + (change in walking) * dt

INIT walking commuters = walkers initial

INFLOWS:

change in walking = ((commuters*mode share walk)-walking commuters)/time to change behaviour

commuters = employed population*commuting fraction

mode share LV = LV utility/total utility

mode share PT = PT utility/total utility

mode share walk = walk utility/total utility

mode share cycle = cycling utility/total utility

time to change behaviour = 1

time to change behaviour = 1

commuting population growth =

employed population(t) = employed population(t - dt) + (Change in worker numbers) * dt

INIT employed population = total workers initial

INFLOWS:

change in worker numbers = employed population*population growth fraction

commuters = employed population*commuting fraction

commuting fraction = 0.85

population growth fraction = GRAPH(TIME)

(1991, 0.045), (1997, 0.023), (2003, 0.03), (2009, 0.011), (2015, 0.011), (2021, 0.012), (2026, 0.013), (2032, 0.012), (2038, 0.012), (2044, 0.012), (2050, 0.012)

light vehicle commuters   initial = 288765

public transport initial = 25464

walkers initial = 18600

total workers initial = 400000

Mode share

cycle commuters(t) = cycle commuters(t - dt) + (change in cycling) * dt

INIT cycle commuters = commuter cyclists initial

INFLOWS:

change in cycling = ((commuters*mode share cycle)-cycle commuters)/time to change behaviour

ASBL on good for work = 0.4*arterial separated bike lanes

ASBL on sense of safety = 0.6*arterial separated bike lanes

cycling utility = prop people considering cycling always or mostly safe*cycling price ok*cycling hassle free*cycling normal*cycling work trip in range*cycling good for work

cycling utility = prop people considering cycling always or mostly safe*cycling price ok*cycling hassle free*cycling normal*cycling work trip in range*cycling good for work

cycling good for work = MIN(1, (init cycling good for work+ASBL on good for work+SER on cycling good for work+RCN on good for work))

cycling hassle free = 0.27

cycling normal = Init cycling normal*Normality in numbers

cycling price ok = 0.95

cycling work trip in range = 0.5

init cycling good for work = 0.1

init cycling normal = 0.7

init cycling safe = 0.22

init lv hassle free = 0.52

LV utility = LV always or mostly safe*LV good for work*LV hassle free*LV normal*LV price ok*LV work trip in range

LV utility = LV always or mostly safe*LV good for work*LV hassle free*LV normal*LV price ok*LV work trip in range

LV hassle free = Init LV hassle free+SER on LV hassle free

LV work trip in range = 1

mode share LV = LV utility/Total utility

mode share PT = PT utility/Total utility

mode share walk = walk utility/Total utility

mode share cycle = cycling utility/Total utility

prop people considering cycling always or mostly safe = MIN(.9,(Init cycling safe*effect of reported injuries on sense of safety)+ASBL on sense of safety+SER on cycling Sense of safety+RCN on sense of safety)

PT utility = PT always or mostly safe*PT good for work*PT hassle free*PT normal*PT price ok*PT work trip in range

PT utility = PT always or mostly safe*PT good for work*PT hassle free*PT normal*PT price ok*PT work trip in range

PT hassle free = 0.24

PT price ok = 0.85

PT work trip in range = 1

RCN on good for work = bus bike lanes good for work+offroad good for work+onroad lanes on good for work

RCN on sense of safety = bus bike lanes sense of safety+offroad sense of safety+onroad lanes sense of safety

reported fatal injuries = Annual fatal serious injuries*.05

response delay = DELAY1(reported fatal injuries,1)

SER on cycling Sense of safety = 0.2*self explaining local roads

SER on cycling good for work = 0.1*Self explaining local roads

SER on LV hassle free = -0.3*Self explaining local roads

total utility = LV utility+cycling utility+PT utility+walk utility

total utility = LV utility+cycling utility+PT utility+walk utility

total utility = LV utility+cycling utility+PT utility+walk utility

total utility = LV utility+cycling utility+PT utility+walk utility

total utility = LV utility+cycling utility+PT utility+walk utility

walk utility = walk always or mostly safe*walk good for work*walk hassle free*walk normal*walk price ok*walk work trip in range

walk utility = walk always or mostly safe*walk good for work*walk hassle free*walk normal*walk price ok*walk work trip in range

walk price ok = 1

walk work trip in range = 0.27

effect of reported injuries on sense of safety = GRAPH(response delay)

(0.00, 1.23), (1.00, 1.18), (2.00, 1.12), (3.00, 0.99), (4.00, 0.81), (5.00, 0.683), (6.00, 0.6), (7.00, 0.532), (8.00, 0.495), (9.00, 0.487), (10.0, 0.48)

LV always or mostly safe = GRAPH(TIME)

(1991, 0.75), (1992, 0.75), (1993, 0.75), (1994, 0.75), (1995, 0.75), (1996, 0.75), (1997, 0.75), (1998, 0.75), (1999, 0.75), (2000, 0.75), (2001, 0.76), (2002, 0.77), (2003, 0.77), (2004, 0.77), (2005, 0.81), (2006, 0.85), (2007, 0.85), (2008, 0.85), (2009, 0.86), (2010, 0.88), (2011, 0.88), (2012, 0.88), (2013, 0.88), (2014, 0.88), (2015, 0.88), (2016, 0.88), (2017, 0.88), (2018, 0.88), (2019, 0.88), (2020, 0.88), (2021, 0.88), (2022, 0.88), (2023, 0.88), (2024, 0.88), (2025, 0.88), (2026, 0.88), (2027, 0.88), (2028, 0.88), (2029, 0.88), (2030, 0.88), (2031, 0.88), (2032, 0.88), (2033, 0.88), (2034, 0.88), (2035, 0.88), (2036, 0.88), (2037, 0.88), (2038, 0.88), (2039, 0.88), (2040, 0.88), (2041, 0.88), (2042, 0.88),

(2043, 0.88), (2044, 0.88), (2045, 0.88), (2046, 0.88), (2047, 0.88), (2048, 0.88), (2049, 0.88), (2050, 0.88)

LV good for work = GRAPH(TIME)

(1991, 0.65), (1992, 0.65), (1993, 0.65), (1994, 0.65), (1995, 0.65), (1996, 0.65), (1997, 0.65), (1998, 0.65), (1999, 0.65), (2000, 0.65), (2001, 0.685), (2002, 0.695), (2003, 0.695), (2004, 0.685), (2005, 0.69), (2006, 0.68), (2007, 0.68), (2008, 0.67), (2009, 0.62), (2010, 0.62), (2011, 0.62), (2012, 0.62), (2013, 0.62), (2014, 0.62), (2015, 0.62), (2016, 0.62), (2017, 0.62), (2018, 0.62), (2019, 0.62), (2020, 0.62), (2021, 0.62), (2022, 0.62), (2023, 0.62), (2024, 0.62), (2025, 0.62), (2026, 0.62), (2027, 0.62), (2028, 0.62), (2029, 0.62), (2030, 0.62), (2031, 0.62), (2032, 0.62), (2033, 0.62), (2034, 0.62), (2035, 0.62), (2036, 0.62), (2037, 0.62), (2038, 0.62), (2039, 0.62), (2040, 0.62), (2041, 0.62), (2042, 0.62), (2043, 0.62), (2044, 0.62), (2045, 0.62), (2046, 0.62), (2047, 0.62), (2048, 0.62), (2049, 0.62), (2050, 0.62)

LV normal = GRAPH(TIME)

(1991, 0.7), (1993, 0.735), (1995, 0.775), (1996, 0.805), (1998, 0.845), (2000, 0.875), (2002, 0.905), (2004, 0.92), (2005, 0.93), (2007, 0.94), (2009, 0.94)

LV price ok = GRAPH(TIME)

(1991, 0.74), (1992, 0.74), (1993, 0.74), (1994, 0.74), (1995, 0.74), (1996, 0.74), (1997, 0.74), (1998, 0.74), (1999, 0.74), (2000, 0.74), (2001, 0.79), (2002, 0.82), (2003, 0.78), (2004, 0.75), (2005, 0.6), (2006, 0.53), (2007, 0.57), (2008, 0.61), (2009, 0.63), (2010, 0.65), (2011, 0.65), (2012, 0.65), (2013, 0.65), (2014, 0.65), (2015, 0.65), (2016, 0.65), (2017, 0.65), (2018, 0.65), (2019, 0.65), (2020, 0.65), (2021, 0.65), (2022, 0.65), (2023, 0.65), (2024, 0.65), (2025, 0.65), (2026, 0.65), (2027, 0.65), (2028, 0.65), (2029, 0.65), (2030, 0.65), (2031, 0.65), (2032, 0.65), (2033, 0.65), (2034, 0.65), (2035, 0.65), (2036, 0.65), (2037, 0.65), (2038, 0.65), (2039, 0.65), (2040, 0.65), (2041, 0.65), (2042, 0.65), (2043, 0.65), (2044, 0.65), (2045, 0.65), (2046, 0.65), (2047, 0.65), (2048, 0.65), (2049, 0.65), (2050, 0.65)

Normality in numbers = GRAPH(Cycle commuters)

(0.00, 0.465), (5000, 0.877), (10000, 1.09), (15000, 1.20), (20000, 1.27), (25000, 1.31), (30000, 1.34), (35000, 1.37), (40000, 1.37), (45000, 1.38), (50000, 1.39), (55000, 1.40), (60000, 1.40), (65000, 1.42), (70000, 1.43), (75000, 1.43), (80000, 1.43), (85000, 1.43), (90000, 1.43), (95000, 1.43), (100000, 1.43)

PT always or mostly safe = GRAPH(TIME)

(1991, 0.62), (1992, 0.62), (1993, 0.62), (1994, 0.62), (1995, 0.62), (1996, 0.62), (1997, 0.62), (1998, 0.62), (1999, 0.62), (2000, 0.62), (2001, 0.62), (2002, 0.62), (2003, 0.63), (2004, 0.63), (2005, 0.69), (2006, 0.69), (2007, 0.69), (2008, 0.68), (2009, 0.75), (2010, 0.75), (2011, 0.75), (2012, 0.75), (2013, 0.75), (2014, 0.75), (2015, 0.75), (2016, 0.75),

(2017, 0.75), (2018, 0.75), (2019, 0.75), (2020, 0.75), (2021, 0.75), (2022, 0.75), (2023, 0.75), (2024, 0.75), (2025, 0.75), (2026, 0.75), (2027, 0.75), (2028, 0.75), (2029, 0.75), (2030, 0.75), (2031, 0.75), (2032, 0.75), (2033, 0.75), (2034, 0.75), (2035, 0.75), (2036, 0.75), (2037, 0.75), (2038, 0.75), (2039, 0.75), (2040, 0.75), (2041, 0.75), (2042, 0.75), (2043, 0.75), (2044, 0.75), (2045, 0.75), (2046, 0.75), (2047, 0.75), (2048, 0.75), (2049, 0.75), (2050, 0.75)

PT good for work = GRAPH(TIME)

(1991, 0.23), (1992, 0.23), (1993, 0.23), (1994, 0.23), (1995, 0.23), (1996, 0.23), (1997, 0.23), (1998, 0.23), (1999, 0.23), (2000, 0.23), (2001, 0.23), (2002, 0.22), (2003, 0.23), (2004, 0.24), (2005, 0.24), (2006, 0.25), (2007, 0.25), (2008, 0.24), (2009, 0.28), (2010, 0.33), (2011, 0.33), (2012, 0.33), (2013, 0.33), (2014, 0.33), (2015, 0.33), (2016, 0.33), (2017, 0.33), (2018, 0.33), (2019, 0.33), (2020, 0.33), (2021, 0.33), (2022, 0.33), (2023, 0.33), (2024, 0.33), (2025, 0.33), (2026, 0.33), (2027, 0.33), (2028, 0.33), (2029, 0.33), (2030, 0.33), (2031, 0.33), (2032, 0.33), (2033, 0.33), (2034, 0.33), (2035, 0.33), (2036, 0.33), (2037, 0.33), (2038, 0.33), (2039, 0.33), (2040, 0.33), (2041, 0.33), (2042, 0.33), (2043, 0.33), (2044, 0.33), (2045, 0.33), (2046, 0.33), (2047, 0.33), (2048, 0.33), (2049, 0.33), (2050, 0.33)

PT normal = GRAPH(TIME)

(1991, 0.355), (1997, 0.36), (2003, 0.375), (2009, 0.39), (2015, 0.395), (2021, 0.41), (2026, 0.435), (2032, 0.46), (2038, 0.49), (2044, 0.52), (2050, 0.545)

walk always or mostly safe = GRAPH(TIME)

(1991, 0.52), (1992, 0.52), (1993, 0.52), (1994, 0.52), (1995, 0.52), (1996, 0.52), (1997, 0.52), (1998, 0.52), (1999, 0.52), (2000, 0.52), (2001, 0.54), (2002, 0.56), (2003, 0.56), (2004, 0.57), (2005, 0.58), (2006, 0.59), (2007, 0.58), (2008, 0.57), (2009, 0.62), (2010, 0.67), (2011, 0.67), (2012, 0.67), (2013, 0.67), (2014, 0.67), (2015, 0.67), (2016, 0.67), (2017, 0.67), (2018, 0.67), (2019, 0.67), (2020, 0.67), (2021, 0.67), (2022, 0.67), (2023, 0.67), (2024, 0.67), (2025, 0.67), (2026, 0.67), (2027, 0.67), (2028, 0.67), (2029, 0.67), (2030, 0.67), (2031, 0.67), (2032, 0.67), (2033, 0.67), (2034, 0.67), (2035, 0.67), (2036, 0.67), (2037, 0.67), (2038, 0.67), (2039, 0.67), (2040, 0.67), (2041, 0.67), (2042, 0.67), (2043, 0.67), (2044, 0.67), (2045, 0.67), (2046, 0.67), (2047, 0.67), (2048, 0.67), (2049, 0.67), (2050, 0.67)

walk good for work = GRAPH(TIME)

(1991, 0.12), (1992, 0.12), (1993, 0.12), (1994, 0.12), (1995, 0.12), (1996, 0.12), (1997, 0.12), (1998, 0.12), (1999, 0.12), (2000, 0.12), (2001, 0.13), (2002, 0.13), (2003, 0.14), (2004, 0.16), (2005, 0.15), (2006, 0.16), (2007, 0.17), (2008, 0.18), (2009, 0.19), (2010, 0.19), (2011, 0.19), (2012, 0.19), (2013, 0.19), (2014, 0.19), (2015, 0.19), (2016, 0.19), (2017, 0.19), (2018, 0.19), (2019, 0.19), (2020, 0.19), (2021, 0.19), (2022, 0.19), (2023, 0.19), (2024, 0.19), (2025, 0.19), (2026, 0.19), (2027, 0.19), (2028, 0.19), (2029, 0.19), (2030, 0.19), (2031, 0.19), (2032, 0.19), (2033, 0.19), (2034, 0.19), (2035, 0.19), (2036,

0.19), (2037, 0.19), (2038, 0.19), (2039, 0.19), (2040, 0.19), (2041, 0.19), (2042, 0.19), (2043, 0.19), (2044, 0.19), (2045, 0.19), (2046, 0.19), (2047, 0.19), (2048, 0.19), (2049, 0.19), (2050, 0.19)

walk hassle free = GRAPH(TIME)

(1991, 0.47), (1992, 0.47), (1993, 0.47), (1994, 0.47), (1995, 0.47), (1996, 0.47), (1997, 0.47), (1998, 0.47), (1999, 0.47), (2000, 0.47), (2001, 0.47), (2002, 0.48), (2003, 0.5), (2004, 0.53), (2005, 0.52), (2006, 0.5), (2007, 0.51), (2008, 0.52), (2009, 0.54), (2010, 0.56), (2011, 0.56), (2012, 0.56), (2013, 0.56), (2014, 0.56), (2015, 0.56), (2016, 0.56), (2017, 0.56), (2018, 0.56), (2019, 0.56), (2020, 0.56), (2021, 0.56), (2022, 0.56), (2023, 0.56), (2024, 0.56), (2025, 0.56), (2026, 0.56), (2027, 0.56), (2028, 0.56), (2029, 0.56), (2030, 0.56), (2031, 0.56), (2032, 0.56), (2033, 0.56), (2034, 0.56), (2035, 0.56), (2036, 0.56), (2037, 0.56), (2038, 0.56), (2039, 0.56), (2040, 0.56), (2041, 0.56), (2042, 0.56), (2043, 0.56), (2044, 0.56), (2045, 0.56), (2046, 0.56), (2047, 0.56), (2048, 0.56), (2049, 0.56), (2050, 0.56)

walk normal = GRAPH(TIME)

(1991, 0.89), (1997, 0.8), (2003, 0.7), (2009, 0.65), (2015, 0.6), (2021, 0.59), (2027, 0.605), (2033, 0.63), (2039, 0.645), (2045, 0.665), (2051, 0.69)

Commuting vehicle kilometres travelled

Light Vehicle Commuters(t) = Light Vehicle Commuters(t - dt) + (change in LV use) * dt

INIT Light Vehicle Commuters = Light vehicle Commuters   initial

INFLOWS:

change in LV use = ((Commuters*mode share LV)-Light Vehicle Commuters)/time to change behaviour

annual fulltime trips = 480

annual part time trips = 240

commute vehicles = Light Vehicle Commuters*vehicles per LV commuter

LV commuting 100mVKT = LV Commuting VKT*1E-008

LV Commuting VKT = ((.21*commute vehicles*annual part time trips)+(commute vehicles*.79*annual fulltime trips))*median LV commute trip length

median LV commute trip length = 6

vehicles per LV commuter = 0.93

Commuter cyclist injury

cumulative fatal serious injuries(t) = cumulative fatal serious injuries(t - dt) + (annual injury rate) * dt

INIT cumulative fatal serious injuries = 0

INFLOWS:

annual injury rate = annual fatal serious injuries

cycle commuters(t) = cycle commuters(t - dt) + (change in cycling) * dt

INIT cycle commuters = Commuter cyclists initial

INFLOWS:

change in cycling = ((commuters*mode share cycle)-cycle commuters)/time to change behaviour

cycle commuters(t) = cycle commuters(t - dt) + (change in cycling) * dt

INIT cycle commuters = commuter cyclists initial

INFLOWS:

change in cycling = ((commuters*mode share cycle)-cycle commuters)/time to change behaviour

other light vehicles(t) = other light vehicles(t - dt) + (vehicle growth) * dt

INIT other light vehicles = other light vehicles initial

INFLOWS:

vehicle growth = other light vehicles*population growth fraction

adapted safety in numbers = 0

adapted SIN = (IF Cycle commuters<17500 THEN 1

ELSE .83^binary log for adapted SIN)*adapted safety in numbers

annual fatal serious injuries = arterial fatal serious injuries+local fatal serious injuries

annual commuter cycle collisions arterial = annual risk of arterial collision per cyclist*arterial commuter cyclists*RR collision ASBL policy*RR collision RCN*RR collision BPI

annual commuter cycle collisions local = annual risk of local collision per cyclist*local commuter cyclists*RR collision SER policy

annual risk of local collision per cyclist = Initial local collision risk per cyclist*SIN effect*Impact of local LV on collisions

annual risk of arterial collision per cyclist = initial arterial collision risk per cyclist*SIN effect*Impact of arterial LV on collisions

arterial commuter cyclists = cycle commuters*proportion cycling arterials

arterial fatal serious injuries = (annual commuter cycle collisions arterial*proportion of arterial collisions fatal or serious)

arterial vehicles = total peak light vehicles*(1-proportion light vehicles local roads)*SER on LV arterial

arterial vehicles = total peak light vehicles*(1-proportion light vehicles local roads)*SER on LV arterial

average arterial LV speed = 30

average local LV speed = (SER local speed*self explaining local roads)+(baseline average car speed local*(1-Self explaining local roads))

baseline average car speed local = 45

binary log for adapted SIN = LN(cycle commuters/(commuters*0.025))/LN(2)

binary log for J SIN = LN(Cycle commuters/commuter cyclists initial)/LN(2)

commuters = employed population*commuting fraction

commuter cyclists initial = 7171

commute vehicles = light vehicle commuters*vehicles per LV commuter

initial arterial collision risk per cyclist = 0.1

Initial local collision risk per cyclist = 0.01

Jacobsen safety in numbers = 0

Jacobsen SIN = Jacobsen safety in numbers*(.66^binary log for J SIN)

local fatal serious injuries = (Annual commuter cycle collisions local*proportion of local collisions fatal or serious)

local commuter cyclists = Cycle commuters*(1-proportion cycling arterials)

local vehicles = total peak light vehicles*proportion light vehicles local roads

local vehicles = total peak light vehicles*proportion light vehicles local roads

proportion cycling arterials = .5*SER on proportion cycling arterial

proportion cycling arterials = .5*SER on proportion cycling arterial

proportion light vehicles local roads = 0.1

proportion of arterial collisions fatal or serious = effect of LV speed on serious injury likelihood 2

proportion of local collisions fatal or serious = effect of LV speed on serious injury likelihood

reported fatal injuries = Annual fatal serious injuries*.05

RR collision BPI = (RR BPI*BPI)+(1-BPI)

RR collision RCN = RR collision RCN lanes*RR collision RCN bus bike lanes*RR collision RCN off-road

RR collision SER policy = (RR collision SER*self explaining local roads)+(1-Self explaining local roads)

RR collision ASBL policy = (arterial separated bike lanes*RR collision ASBL)+(1-arterial separated bike lanes)

self explaining local roads = implement SER policy in 2012*SER implementation

serious injury rate per 1000 cyclists = 1000*(Annual fatal serious injuries/cycle commuters)

SER on LV arterial = (self explaining local roads*.75)+(1-self explaining local roads)

SER on proportion cycling arterial = (self explaining local roads*.6)+(1-self explaining local roads)

SER local speed = 30

SIN effect = adapted SIN+Jacobsen SIN

total peak light vehicles = commute vehicles+other light vehicles

effect of LV speed on serious injury likelihood = GRAPH(average local LV speed)

(0.00, 0.00), (5.00, 0.005), (10.0, 0.01), (15.0, 0.02), (20.0, 0.03), (25.0, 0.05), (30.0, 0.075), (35.0, 0.11), (40.0, 0.145), (45.0, 0.2), (50.0, 0.28), (55.0, 0.435), (60.0, 0.71), (65.0, 0.89), (70.0, 0.95), (75.0, 0.98), (80.0, 1.00), (85.0, 1.00), (90.0, 1.00), (95.0, 1.00), (100, 1.00)

effect of LV speed on serious injury likelihood 2 = GRAPH(average arterial LV speed)

(0.00, 0.00), (5.00, 0.005), (10.0, 0.01), (15.0, 0.02), (20.0, 0.03), (25.0, 0.05), (30.0, 0.075), (35.0, 0.11), (40.0, 0.145), (45.0, 0.2), (50.0, 0.28), (55.0, 0.435), (60.0, 0.71), (65.0, 0.9), (70.0, 0.95), (75.0, 0.98), (80.0, 1.00), (85.0, 1.00), (90.0, 1.00), (95.0, 1.00), (100, 1.00)

impact of arterial LV on collisions = GRAPH(Arterial vehicles)

(100000, 0.25), (160000, 0.45), (220000, 0.68), (280000, 0.87), (340000, 0.97), (400000, 1.00), (460000, 1.02), (520000, 1.03), (580000, 1.04), (640000, 1.05), (700000, 1.05)

impact of local LV on collisions = GRAPH(local vehicles)

(0.00, 0.00), (15000, 0.333), (30000, 0.666), (45000, 1.00), (60000, 1.33), (75000, 1.67), (90000, 2.00), (105000, 2.33), (120000, 2.67), (135000, 3.00), (150000, 3.33)

population growth fraction = GRAPH(TIME)

(1991, 0.045), (1997, 0.023), (2003, 0.03), (2009, 0.011), (2015, 0.011), (2021, 0.012), (2026, 0.013), (2032, 0.012), (2038, 0.012), (2044, 0.012), (2050, 0.012)

commuter cyclist injuries per year initial = 168

commute vehicles initial = Light vehicle Commuters initial*vehicles per LV commuter

other light vehicles initial = commute vehicles initial*(1-Proportion LV commuting)/Proportion LV commuting

Air pollution outcomes

cumulative air pollution hospitalisations(t) = cumulative air pollution hospitalisations(t - dt) + (hospitalisation rate) * dt

INIT cumulative air pollution hospitalisations = 0

INFLOWS:

hospitalisation rate = annual air pollution hospitalisations

cumulative air pollution mortality(t) = cumulative air pollution mortality(t - dt) + (AP mortality rate) * dt

INIT cumulative air pollution mortality = 0

INFLOWS:

AP mortality rate = annual air pollution mortality

cumulative COPD incidence(t) = cumulative COPD incidence(t - dt) + (COPD rate) * dt

INIT cumulative COPD incidence = 0

INFLOWS:

COPD rate = annual COPD incidence

cumulative new cancers(t) = cumulative new cancers(t - dt) + (cancer rate) * dt

INIT cumulative new cancers = 0

INFLOWS:

cancer rate = annual new cancer

cumulative RAD(t) = cumulative RAD(t - dt) + (RAD rate) * dt

INIT cumulative RAD = 0

INFLOWS:

RAD rate = annual RAD

annual air pollution hospitalisations = pop adj hosp per 100mvkt*LV commuting 100mVKT

annual air pollution hospitalisations = pop adj hosp per 100mvkt*LV commuting 100mVKT

annual air pollution mortality = pop adj mortality per 100mvkt*LV commuting 100mVKT

annual air pollution mortality = pop adj mortality per 100mvkt*LV commuting 100mVKT

annual COPD incidence = LV commuting 100mVKT*pop adj COPD per 100mvkt

annual COPD incidence = LV commuting 100mVKT*pop adj COPD per 100mvkt

annual new cancer = pop adj cancer per 100mvkt*LV commuting 100mVKT

annual new cancer = pop adj cancer per 100mvkt*LV commuting 100mVKT

annual RAD = LV commuting 100mVKT*pop adj RAD per 100mvkt

annual RAD = LV commuting 100mVKT*pop adj RAD per 100mvkt

cancer per 100m vkt 1991 = 0.0015

COPD per 100m vkt 1991 = 2.212

hosp per 100m vkt 1991 = 1.007

LV commuting 100mVKT = LV Commuting VKT*1E-008

mortality per 100m vkt 1991 = 1.854

pop adj cancer per 100mvkt = cancer per 100m vkt 1991*light fleet PM10 emission improvement*total popn growth

pop adj COPD per 100mvkt = COPD per 100m vkt 1991*light fleet PM10 emission improvement*total popn growth

pop adj hosp per 100mvkt = hosp per 100m vkt 1991*light fleet PM10 emission improvement*total popn growth

pop adj mortality per 100mvkt = mortality per 100m vkt 1991*light fleet PM10 emission improvement*total popn growth

pop adj RAD per 100mvkt = RAD per 100m vkt 1991*light fleet PM10 emission improvement*total popn growth

RAD per 100m vkt 1991 = 2746.077

light fleet PM10 emission improvement = GRAPH(TIME)

(1991, 1.20), (1994, 1.19), (1997, 1.14), (2000, 1.07), (2003, 0.99), (2006, 0.877), (2009, 0.72), (2012, 0.593), (2015, 0.472), (2018, 0.39), (2021, 0.315), (2024, 0.277), (2027, 0.255), (2030, 0.25), (2033, 0.24), (2036, 0.24), (2039, 0.24), (2042, 0.23), (2045, 0.23), (2048, 0.23), (2051, 0.23)

light fleet PM10 emission improvement = GRAPH(TIME)

(1991, 1.20), (1994, 1.19), (1997, 1.14), (2000, 1.07), (2003, 0.99), (2006, 0.877), (2009, 0.72), (2012, 0.593), (2015, 0.472), (2018, 0.39), (2021, 0.315), (2024, 0.277), (2027, 0.255), (2030, 0.25), (2033, 0.24), (2036, 0.24), (2039, 0.24), (2042, 0.23), (2045, 0.23), (2048, 0.23), (2051, 0.23)

light fleet PM10 emission improvement = GRAPH(TIME)

(1991, 1.20), (1994, 1.19), (1997, 1.14), (2000, 1.07), (2003, 0.99), (2006, 0.877), (2009, 0.72), (2012, 0.593), (2015, 0.472), (2018, 0.39), (2021, 0.315), (2024, 0.277), (2027, 0.255), (2030, 0.25), (2033, 0.24), (2036, 0.24), (2039, 0.24), (2042, 0.23), (2045, 0.23), (2048, 0.23), (2051, 0.23)

total popn growth = GRAPH(TIME)

(1991, 1.01), (1997, 1.01), (2003, 1.03), (2009, 1.03), (2015, 1.01), (2021, 1.01), (2026, 1.01), (2032, 1.01), (2038, 1.01), (2044, 1.01), (2050, 1.01)

total popn growth = GRAPH(TIME)

(1991, 1.01), (1997, 1.01), (2003, 1.03), (2009, 1.03), (2015, 1.01), (2021, 1.01), (2026, 1.01), (2032, 1.01), (2038, 1.01), (2044, 1.01), (2050, 1.01)

total popn growth = GRAPH(TIME)

(1991, 1.01), (1997, 1.01), (2003, 1.03), (2009, 1.03), (2015, 1.01), (2021, 1.01), (2026, 1.01), (2032, 1.01), (2038, 1.01), (2044, 1.01), (2050, 1.01)

Physical activity related mortality

cumulative stratified mortality[ethnicity and gender, age group](t) = cumulative stratified mortality[ethnicity and gender, age group](t - dt) + (all cause mortality accumulation[ethnicity and gender, age group]) * dt

INIT cumulative stratified mortality[ethnicity and gender, age group] = 0

INFLOWS:

all cause mortality accumulation[ethnicity and gender, age group] = commuter mortality accounting for cycling and lead in[ethnicity and gender,age group]

cumulative total mortality savings(t) = cumulative total mortality savings(t - dt) + (mortality rate) * dt

INIT cumulative total mortality savings = 0

INFLOWS:

mortality rate = total mortality savings due to intervention

backcasting adjustment for mortality trends = 1.05

baseline 1991 all cause mortality rates[ethnicity and gender, age group] = expected mortality rates no cycling 1996[ethnicity and gender,age group]*backcasting adjustment for mortality trends

commuters = employed population*commuting fraction

commuters by age ethnicity gender[ethnicity and gender, age group] = commuters*prop commuters by age ethnicity gender 1996[ethnicity and gender,age group]

commuter mortality accounting for cycling[ethnicity and gender, age group] = (mortality rates accounting for cycling[ethnicity and gender,age group]*commuters by age ethnicity gender[ethnicity and gender,age group])/1000

commuter mortality accounting for cycling and lead in[ethnicity and gender, age group] = DELAY3(commuter mortality accounting for cycling[ethnicity and gender,age group], lead in and out time)

cycling RR all cause mort = 0.72

expected mortality rates no cycling 1996[ethnicity and gender, age group] = standardised national mortality rates 1996[ethnicity and gender,age group]/0.987

lead in and out time = 2

mode share cycle = cycling utility/total utility

mortality rates accounting for cycling[ethnicity and gender, age group] = (trend adj expected all cause mortality rates no cycling[ethnicity and gender,age group]*(1-mode share cycle))+(trend adj expected all cause mortality rates no cycling[ethnicity and gender,age group]*cycling RR all cause mort*mode share cycle)

prop commuters by age ethnicity gender 1996[Maori M, a15 to 24] = 0.0151

prop commuters by age ethnicity gender 1996[Maori M, b25 to 44] = 0.0312

prop commuters by age ethnicity gender 1996[Maori M, c 45 to 64] = 0.0099

prop commuters by age ethnicity gender 1996[Maori M, d 65 and over] = 0.0002

prop commuters by age ethnicity gender 1996[Maori F, a15 to 24] = 0.0124

prop commuters by age ethnicity gender 1996[Maori F, b25 to 44] = 0.0235

prop commuters by age ethnicity gender 1996[Maori F, c 45 to 64] = 0.0080

prop commuters by age ethnicity gender 1996[Maori F, d 65 and over] = 0.0001

prop commuters by age ethnicity gender 1996[Pacific M, a15 to 24] = 0.0109

prop commuters by age ethnicity gender 1996[Pacific M, b25 to 44] = 0.0273

prop commuters by age ethnicity gender 1996[Pacific M, c 45 to 64] = 0.0079

prop commuters by age ethnicity gender 1996[Pacific M, d 65 and over] = 0.0001

prop commuters by age ethnicity gender 1996[Pacific F, a15 to 24] = 0.0096

prop commuters by age ethnicity gender 1996[Pacific F, b25 to 44] = 0.0214

prop commuters by age ethnicity gender 1996[Pacific F, c 45 to 64] = 0.0064

prop commuters by age ethnicity gender 1996[Pacific F, d 65 and over] = 0.0001

prop commuters by age ethnicity gender 1996[Other M, a15 to 24] = 0.0717

prop commuters by age ethnicity gender 1996[Other M, b25 to 44] = 0.2423

prop commuters by age ethnicity gender 1996[Other M, c 45 to 64] = 0.1421

prop commuters by age ethnicity gender 1996[Other M, d 65 and over] = 0.0076

prop commuters by age ethnicity gender 1996[Other F, a15 to 24] = 0.0670

prop commuters by age ethnicity gender 1996[Other F, b25 to 44] = 0.1786

prop commuters by age ethnicity gender 1996[Other F, c 45 to 64] = 0.1040

prop commuters by age ethnicity gender 1996[Other F, d 65 and over] = 0.0027

standardised national mortality rates 1996[Maori M, a15 to 24] = 1.7

standardised national mortality rates 1996[Maori M, b25 to 44] = 2.8

standardised national mortality rates 1996[Maori M, c 45 to 64] = 15.9

standardised national mortality rates 1996[Maori M, d 65 and over] = 48

standardised national mortality rates 1996[Maori F, a15 to 24] = .7

standardised national mortality rates 1996[Maori F, b25 to 44] = 1.6

standardised national mortality rates 1996[Maori F, c 45 to 64] = 11.3

standardised national mortality rates 1996[Maori F, d 65 and over] = 33.8

standardised national mortality rates 1996[Pacific M, a15 to 24] = 1.2

standardised national mortality rates 1996[Pacific M, b25 to 44] = 2

standardised national mortality rates 1996[Pacific M, c 45 to 64] = 11.7

standardised national mortality rates 1996[Pacific M, d 65 and over] = 41.3

standardised national mortality rates 1996[Pacific F, a15 to 24] = .4

standardised national mortality rates 1996[Pacific F, b25 to 44] = 1.5

standardised national mortality rates 1996[Pacific F, c 45 to 64] = 7.6

standardised national mortality rates 1996[Pacific F, d 65 and over] = 21.5

standardised national mortality rates 1996[Other M, a15 to 24] = 1.2

standardised national mortality rates 1996[Other M, b25 to 44] = 1.3

standardised national mortality rates 1996[Other M, c 45 to 64] = 6

standardised national mortality rates 1996[Other M, d 65 and over] = 22.2

standardised national mortality rates 1996[Other F, a15 to 24] = .5

standardised national mortality rates 1996[Other F, b25 to 44] = .6

standardised national mortality rates 1996[Other F, c 45 to 64] = 3.9

standardised national mortality rates 1996[Other F, d 65 and over] = 12.7

TOTAL annual commuter mortality = SUM(commuter mortality accounting for cycling and lead in)

total mortality savings due to intervention = TOTAL BAU mortality-TOTAL annual commuter mortality

trend adj expected all cause mortality rates no cycling[ethnicity and gender, age group] = baseline 1991 all cause mortality rates[ethnicity and gender,age group]*Adjustment for mortality trends

adjustment for mortality trends = GRAPH(TIME)

(1991, 1.00), (1997, 0.96), (2003, 0.93), (2009, 0.91), (2015, 0.88), (2021, 0.86), (2026, 0.82), (2032, 0.8), (2038, 0.79), (2044, 0.76), (2050, 0.74)

TOTAL BAU mortality = GRAPH(TIME)

(1991, 961), (1991, 961), (1992, 962), (1992, 964), (1992, 967), (1992, 971), (1993, 975), (1993, 980), (1993, 986), (1993, 991), (1994, 997), (1994, 1004), (1994, 1010), (1994, 1016), (1995, 1023), (1995, 1030), (1995, 1036), (1995, 1043), (1996, 1050), (1996, 1056), (1996, 1063), (1996, 1069), (1997, 1075), (1997, 1082), (1997, 1088), (1997, 1093), (1998, 1099), (1998, 1105), (1998, 1110), (1998, 1116), (1999, 1122), (1999, 1127), (1999, 1133), (1999, 1138), (2000, 1144), (2000, 1150), (2000, 1156), (2000, 1161), (2001, 1167), (2001, 1173), (2001, 1180), (2001, 1186), (2002, 1192), (2002, 1198), (2002, 1205), (2002, 1212), (2003, 1218), (2003, 1225), (2003, 1232), (2003, 1239), (2004, 1246), (2004, 1254), (2004, 1261), (2004, 1269), (2005, 1276), (2005, 1283), (2005, 1291), (2005, 1298), (2006, 1305), (2006, 1312), (2006, 1319), (2006, 1325), (2007, 1332), (2007, 1338), (2007, 1344), (2007, 1350), (2008, 1356), (2008, 1362), (2008, 1367), (2008, 1373), (2009, 1378), (2009, 1382), (2009, 1387), (2009, 1391), (2010, 1395), (2010, 1398), (2010, 1402), (2010, 1405), (2011, 1408), (2011, 1411), (2011, 1414), (2011, 1417), (2012, 1419), (2012, 1422), (2012, 1424), (2012, 1427), (2013, 1429), (2013, 1431), (2013, 1434), (2013, 1436), (2014, 1438), (2014, 1440), (2014, 1442), (2014, 1444), (2015, 1446), (2015, 1448), (2015, 1450), (2015, 1453), (2016, 1455), (2016, 1457), (2016, 1459), (2016, 1462), (2017, 1464), (2017, 1466), (2017, 1469), (2017, 1472), (2018, 1474), (2018, 1477), (2018, 1479), (2018, 1482), (2019, 1485), (2019, 1487), (2019, 1490), (2019, 1493), (2020, 1496), (2020, 1498), (2020, 1501), (2020, 1504), (2021, 1507), (2021, 1510), (2021, 1513), (2021, 1515), (2022, 1518), (2022, 1520), (2022, 1522), (2022, 1524), (2023, 1526), (2023, 1529), (2023, 1531), (2023, 1532), (2024, 1534), (2024, 1536), (2024, 1538), (2024, 1540), (2025, 1542), (2025, 1544), (2025, 1546), (2025, 1547), (2026, 1549), (2026, 1551), (2026, 1553), (2026, 1555), (2027, 1556), (2027, 1558), (2027, 1560), (2027, 1563), (2028, 1565), (2028, 1568), (2028, 1570), (2028, 1573), (2029, 1576), (2029, 1579), (2029, 1582), (2029, 1585), (2030, 1588), (2030, 1591), (2030, 1594), (2030, 1597), (2031, 1600), (2031, 1603), (2031, 1607), (2031, 1610), (2032, 1613), (2032, 1616), (2032, 1619), (2032, 1623), (2033, 1626), (2033, 1629), (2033, 1633), (2033, 1636), (2034, 1640), (2034, 1643), (2034, 1647), (2034, 1651), (2035, 1654), (2035, 1658), (2035, 1662), (2035, 1666), (2036, 1670), (2036, 1674), (2036, 1678), (2036, 1682), (2037, 1686), (2037, 1690), (2037, 1694), (2037, 1698), (2038, 1703), (2038, 1707), (2038, 1711), (2038, 1715), (2039, 1719), (2039, 1723), (2039, 1727), (2039, 1730), (2040, 1734), (2040, 1737), (2040, 1740), (2040, 1743), (2041, 1746), (2041, 1749), (2041, 1752), (2041, 1755), (2042, 1758), (2042, 1761), (2042, 1763), (2042, 1766), (2043, 1768), (2043, 1771), (2043, 1774), (2043, 1776), (2044, 1779), (2044, 1781), (2044, 1784), (2044, 1786), (2045, 1789), (2045, 1791), (2045, 1794), (2045, 1797), (2046, 1800), (2046, 1803), (2046, 1806), (2046, 1809),

(2047, 1812), (2047, 1815), (2047, 1818), (2047, 1822), (2048, 1825), (2048, 1828), (2048, 1831), (2048, 1835), (2049, 1838), (2049, 1841), (2049, 1845), (2049, 1848), (2050, 1852), (2050, 1855), (2050, 1858), (2050, 1862), (2051, 1865), (2051, 1869), (2051, 1874)

Fuel costs

cumulative fuel cost savings(t) = cumulative fuel cost savings(t - dt) + (fuel cost saving rate) * dt

INIT cumulative fuel cost savings = 0

INFLOWS:

fuel cost saving rate = Total savings 2008 million NZD

cycle commuters(t) = cycle commuters(t - dt) + (change in cycling) * dt

INIT cycle commuters = commuter cyclists initial

INFLOWS:

change in cycling = ((commuters*mode share cycle)-cycle commuters)/time to change behaviour

cycle commuters(t) = cycle commuters(t - dt) + (change in cycling) * dt

INIT cycle commuters = commuter cyclists initial

INFLOWS:

change in cycling = ((commuters*mode share cycle)-cycle commuters)/time to change behaviour

annual fulltime trips = 480

annual fulltime trips = 480

annual part time trips = 240

annual part time trips = 240

average LVKT per commuter = vehicles per LV commuter*((.21*annual part time trips)+(.79*annual fulltime trips))*median LV commute trip length

diesel saved = LVKT savings diesel*Fuel consumption

LVKT saved by cyclists = cycle commuters*average LVKT per commuter

LVKT savings diesel = LVKT saved by cyclists*(1-prop LV petrol)

LVKT savings petrol = LVKT saved by cyclists*prop LV petrol

median LV commute trip length = 6

petrol saved = LVKT savings petrol*Fuel consumption

Saving per cyclist = 10^6*Total savings 2008 million NZD/cycle commuters

saving per trip = Saving per cyclist/((.21*annual part time trips)+(.79*annual fulltime trips))

Total savings 2008 million NZD = ((petrol saved*price petrol)+(diesel saved*price diesel))/10^6

vehicles per LV commuter = 0.93

Fuel consumption = GRAPH(TIME)

(1991, 0.116), (1997, 0.115), (2003, 0.111), (2009, 0.109), (2015, 0.104), (2021, 0.094), (2026, 0.084), (2032, 0.076), (2038, 0.071), (2044, 0.066), (2050, 0.065)

price diesel = GRAPH(TIME)

(1991, 0.5), (1997, 0.6), (2003, 0.5), (2009, 1.59), (2015, 2.13), (2021, 2.13), (2027, 2.58), (2033, 2.94), (2039, 3.37), (2045, 3.98), (2051, 4.73)

price petrol = GRAPH(TIME)

(1991, 0.97), (1997, 0.9), (2003, 1.09), (2009, 2.03), (2015, 2.57), (2021, 2.57), (2027, 3.02), (2033, 3.25), (2039, 3.38), (2045, 4.42), (2051, 5.17)

prop LV petrol = GRAPH(TIME)

(1991, 0.88), (1997, 0.865), (2003, 0.837), (2009, 0.799), (2015, 0.775), (2021, 0.704), (2026, 0.595), (2032, 0.51), (2038, 0.415), (2044, 0.365), (2050, 0.34)

CPI adjusted petrol price = GRAPH(TIME)

(1991, 96.7), (1996, 86.5), (2001, 92.0), (2006, 113)

Greenhouse gas emissions

cumulative CO2eqMt savings(t) = cumulative CO2eqMt savings(t - dt) + (CO2eq accumulation) * dt

INIT cumulative CO2eqMt savings = 0

INFLOWS:

CO2eq accumulation = annual commuting CO2eq M t

annual CO2eq = LV commuting 100mVKT*LV fleet trend CO2eq emissions*initial CO2eq per 100mLVKT

annual commuting CO2eq M t = annual CO2eq*10E-6

initial CO2eq per 100mLVKT = 27642

LV commuting 100mVKT = LV Commuting VKT*1E-008

per capita CO2eq t = annual commuting CO2eq M t*1000000/(total Ak pop 1991*total popn growth)

total Ak pop 1991 = 943773

LV fleet trend CO2eq emissions = GRAPH(TIME)

(1991, 1.00), (1997, 1.00), (2003, 0.997), (2009, 0.974), (2015, 0.919), (2021, 0.838), (2026, 0.747), (2032, 0.652), (2038, 0.555), (2044, 0.495), (2050, 0.45)

total popn growth = GRAPH(TIME)

(1991, 1.01), (1997, 1.01), (2003, 1.03), (2009, 1.03), (2015, 1.01), (2021, 1.01), (2026, 1.01), (2032, 1.01), (2038, 1.01), (2044, 1.01), (2050, 1.01)

RLTS 2010 Regional Cycle Network

arterial onroad lanes = Complete the RLTS cycle network*complete onroad lanes

bus bike lanes 3m = 0

bus bike lanes 4'5m = 0

bus bike lanes default = 1

bus bike lanes good for work = .2*Shared bus bike lanes

bus bike lanes sense of safety = 0*Shared bus bike lanes

complete the RLTS cycle network = 0

km arterial road Auckland = 1287

offroad good for work = 0.1*off road tracks

offroad sense of safety = 0.5*off road tracks

offroad tracks = complete the RLTS cycle network*complete offroad tracks

onroad lanes sense of safety = 0.4*arterial onroad lanes

onroad lanes on good for work = 0.3*arterial onroad lanes

RCN on good for work = bus bike lanes good for work+offroad good for work+onroad lanes on good for work

RCN on sense of safety = bus bike lanes sense of safety+offroad sense of safety+onroad lanes sense of safety

RR 3m = 2.2*bus bike lanes 3m

RR 4'5m = .5*bus bike lanes 4'5m

RR collision bus bike lane = RR default+RR 3m+RR 4'5m

RR collision RCN = RR collision RCN lanes*RR collision RCN bus bike lanes*RR collision RCN offroad

RR collision RCN bus bike lanes = (RR collision bus bike lane*Shared bus bike lanes)+(1-Shared bus bike lanes)

RR collision RCN offroad = (RR collision offroad tracks*off road tracks)+(1-off road tracks)

RR collision offroad tracks = 1

RR collision onroad lanes = 0.9

RR collision RCN lanes = (RR collision onroad lanes*arterial onroad lanes)+(1-arterial onroad lanes)

RR default = bus bike lanes default

shared bus bike lanes = complete the RLTS cycle network*complete bus bike lanes

total km bus bike lane = shared bus bike lanes*km arterial road Auckland

total km offroad track = off road tracks*km arterial road Auckland

total onroad lane km = arterial onroad lanes*km arterial road Auckland

complete bus bike lanes = GRAPH(TIME)

(1991, 0.00), (1997, 0.00), (2003, 0.00), (2009, 0.00), (2015, 0.01), (2021, 0.02), (2027, 0.025), (2033, 0.027), (2039, 0.03), (2045, 0.03), (2051, 0.03)

complete offroad tracks = GRAPH(TIME)

(1991, 0.00), (1997, 0.00), (2003, 0.00), (2009, 0.01), (2015, 0.03), (2021, 0.075), (2027, 0.135), (2033, 0.155), (2039, 0.17), (2045, 0.17), (2051, 0.17)

complete onroad lanes = GRAPH(TIME)

(1991, 0.00), (1997, 0.00), (2003, 0.00), (2009, 0.005), (2015, 0.05), (2021, 0.14), (2027, 0.315), (2033, 0.41), (2039, 0.45), (2045, 0.46), (2051, 0.46)

ASBL Policy

arterial separated bikelanes = Implement ASBL policy in 2012*ASBL implementation

ASBL on good for work = 0.4*arterial separated bikelanes

ASBL on sense of safety = 0.6*arterial separated bikelanes

best practice intersections = 0

BPI = best practice intersections*ASBL implementation

BPI on sense of safety = (0.1*BPI)+(1-BPI)

implement ASBL policy in 2012 = 0

RR BPI = 0.75

RR collision BPI = (RR BPI*BPI)+(1-BPI)

RR collision ASBL policy = (arterial separated bikelanes*RR collision ASBL)+(1-arterial separated bikelanes)

RR collision ASBL = 0.72

ASBL implementation = GRAPH(TIME)

(1991, 0.00), (1997, 0.00), (2003, 0.00), (2009, 0.00), (2015, 0.05), (2021, 0.215), (2026, 0.68), (2032, 0.865), (2038, 0.94), (2044, 0.96), (2050, 0.975)

ASBL implementation = GRAPH(TIME)

(1991, 0.00), (1997, 0.00), (2003, 0.00), (2009, 0.00), (2015, 0.05), (2021, 0.215), (2026, 0.68), (2032, 0.865), (2038, 0.94), (2044, 0.96), (2050, 0.975)

SER Policy

implement SER policy in 2012 = 0

RR collision SER = 0.4

RR collision SER policy = (RR collision SER*self explaining local roads)+(1-Self explaining local roads)

self explaining local roads = Implement SER policy in 2012*SER implementation

SER on cycling Sense of safety = 0.2*Self explaining local roads

SER on cycling good for work = 0.1*self explaining local roads

SER on LV hassle free = -0.3*self explaining local roads

SER on LV arterial = (self explaining local roads*.75)+(1-Self explaining local roads)

SER on proportion cycling arterial = (self explaining local roads*.6)+(1-Self explaining local roads)

SER local speed = 30

SER implementation = GRAPH(TIME)

(1991, 0.00), (1997, 0.00), (2003, 0.00), (2009, 0.00), (2015, 0.035), (2021, 0.175), (2026, 0.68), (2032, 0.865), (2038, 0.94), (2044, 0.96), (2050, 0.975)

Intervention costs

cost per km ASBL(t) = cost per km ASBL(t - dt) + (escalation asbl) * dt

INIT cost per km ASBL = 200000

INFLOWS:

escalation ASBL = escalation rate*cost per km ASBL

cost per km bus bike lane(t) = cost per km bus bike lane(t - dt) + (escalation bb) * dt

INIT cost per km bus bike lane = 100000

INFLOWS:

escalation bus bike lane = cost per km bus bike lane*escalation rate

cost per km offroad track(t) = cost per km offroad track(t - dt) + (escalation ot) * dt

INIT cost per km offroad track = 150000

INFLOWS:

escalation ot = escalation rate*cost per km offroad track

cost per km onroad lane(t) = cost per km onroad lane(t - dt) + (escalation ol) * dt

INIT cost per km onroad lane = 15000

INFLOWS:

escalation ol = escalation rate*cost per km onroad lane

cost per km SER(t) = cost per km SER(t - dt) + (escalation ser) * dt

INIT cost per km SER = 300000

INFLOWS:

escalation SER = escalation rate*cost per km SER

arterial separated bike lanes = Implement ASBL policy in 2012*ASBL implementation

average annual cost RCN = cost of RCN to 2050/time to implement RCN

average annual cost RCN = cost of RCN to 2050/time to implement RCN

average annual cost ASBL = cost of ASBL to 2050/time to implement ASBL

average annual cost ASBL = cost of ASBL to 2050/time to implement ASBL

average annual cost SER = cost of SER to 2050/time to implement SER

average annual cost SER = cost of SER to 2050/time to implement SER

cost bus bike lanes to 2051 = cost per km bus bike lane*total km bus bike lane

cost of RCN to 2050 = (cost bus bike lanes to 2051+cost offroad track to 2050+cost onroad lanes to 2050)/1000000

cost of RCN to 2050 = (cost bus bike lanes to 2051+cost offroad track to 2050+cost onroad lanes to 2050)/1000000

cost offroad track to 2050 = cost per km offroad track*total km offroad track

cost of ASBL to 2050 = (arterial separated bikelanes*cost per km ASBL*km arterial road Auckland)/1000000

cost of ASBL to 2050 = (arterial separated bikelanes*cost per km ASBL*km arterial road Auckland)/1000000

cost of SER to 2050 = (cost per km SER*km local road Auckland*self explaining local roads)/1000000

cost of SER to 2050 = (cost per km SER*km local road Auckland*self explaining local roads)/1000000

cost onroad lanes to 2050 = cost per km onroad lane*total onroad lane km

escalation rate = 0.03

escalation rate = 0.03

escalation rate = 0.03

km arterial road Auckland = 1287

km local road Auckland = 1296

self explaining local roads = Implement SER policy in 2012*SER implementation

time to implement ASBL = 39

time to implement RCN = 29

time to implement SER = 39

total cost million = (cost of ASBL to 2050 + Cost of SER to 2050 + cost of RCN to 2050)

total annual cost million = (average annual cost ASBL + average annual cost SER + average annual cost RCN)

total km bus bike lane = Shared bus bike lanes*km arterial road Auckland

total km offroad track = off road tracks*km arterial road Auckland

total onroad lane km = arterial onroad lanes*km arterial road Auckland

References

[Anonymous]. 2003. Land transport management act. New Zealand Government. http://www.legislation.govt.nz/act/public/2003/0118/latest/viewpdf.aspx

Barlas Y. 1996. Formal aspects of model validity and validation in system dynamics. System Dynamics Review 12:183-210. http://dx.doi.org/10.1002/(SICI)1099-1727(199623)12:3<183::AID-SDR103>3.0.CO;2-4

Forrester JW, Senge PM. 1980. Tests for building confidence in system dynamics models. In: System dynamics, (Legasto Jr A, Forrester JW, Lyneis J, eds). Amsterdam:North-Holland 209-228.

Sterman JD. 2000. Business dynamics. Systems thinking and modeling for a complex world. Boston:Irwin McGraw-Hill.

van den Belt M. 2004. Mediated modelling. A system dynamics approach to environmental consensus building. Washington:Island Press.
